# Supplementary material for: Adsorption energies on transition metal surfaces: towards an accurate and balanced description
Source: Nat Commun. 2022 Nov 11;13:6853. doi: 10.1038/s41467-022-34507-y (PMC9652424; doi:10.1038/s41467-022-34507-y)
Supplement: Supplementary file 1 — Supplementary Information [file 41467_2022_34507_MOESM1_ESM.pdf]

Supplementary Information for: 'Adsorption Energies on Transition Metals Surfaces: Towards an Accurate and Balanced Description'

Araujo et al.

Supplementary Note 1

Optimized structure of C_6H_6/Pt(111)

C_6H_6_Pt111:
27
Cluster structure (xyz format; Ångström)

Pt	12.53169669	8.81951902	7.11736263
Pt	13.90533434	11.21480254	7.15698980
Pt	12.48884767	12.07730979	9.38406973
Pt	13.92183100	6.42921244	7.13021967
Pt	16.66276914	6.43025807	7.12996328
Pt	15.29281937	8.83729397	7.16332612
Pt	18.05564930	8.82036605	7.11689501
Pt	16.68271269	11.21636789	7.15290701
Pt	12.49213394	7.18169737	9.38061056
Pt	15.30951171	7.25914956	9.52679642
Pt	18.12099113	7.17993712	9.37831653
Pt	13.82455157	9.64465876	9.49357908
Pt	16.79304075	9.64644165	9.48568560
Pt	15.30698514	12.02064527	9.55368308
Pt	18.12432374	12.08135360	9.38560696
H	13.17074983	10.84643478	11.87005820
H	15.32794246	7.20056959	12.12139794
C	15.31901108	11.13320334	11.51893416
C	16.55831547	10.33835534	11.54882127
C	16.56119858	8.90430019	11.53999538
C	15.32344827	8.10676746	11.50564540
C	14.08364606	8.90041487	11.54715233
C	14.08173859	10.33502836	11.55424410
H	15.32062262	12.03354610	12.14382602
H	17.47152519	10.85162781	11.85587481
H	17.47658637	8.39113017	11.84201429
H	13.17309622	8.38345289	11.85643229

C_6H_6/Pt111 structure with 5 Pt layers slab (POSCAR format):

```
Pt  H  C  H
   1.00000000000000
     8.3367889501893959    0.0000000000000000    0.0000000000000000
     4.1683944750946980    7.2198710168534168    0.0000000000000000
     0.0000000000000000    0.0000000000000000   23.0759462316609181
   Pt   H    C    H
    45    2    6    4
Selective dynamics
Direct
  0.9144764033372326  0.1934946254310006  0.1216057653510703   F   F   F
  0.2478097366705683  0.1934946254310006  0.1216057653510703   F   F   F
  0.5811430700038969  0.1934946254310006  0.1216057653510703   F   F   F
  0.9144764033372326  0.5268279587643363  0.1216057653510703   F   F   F
  0.2478097366705683  0.5268279587643363  0.1216057653510703   F   F   F
  0.5811430700038969  0.5268279587643363  0.1216057653510703   F   F   F
  0.9144764033372326  0.8601612920976649  0.1216057653510703   F   F   F
```

0.2478097366705683	0.8601612920976649	0.1216057653510703	F	F	F
0.5811430700038969	0.8601612920976649	0.1216057653510703	F	F	F
0.0228962480068147	0.9672568285081866	0.2208079034827217	T	T	T
0.3564030041337661	0.9670467592941380	0.2217260400500299	T	T	T
0.6897077473349090	0.9672865947196773	0.2207528800914902	T	T	T
0.0232823780059122	0.3004443416119634	0.2218152080346320	T	T	T
0.3560017901433476	0.3004609108205153	0.2217626296846819	T	T	T
0.6896488441254860	0.3007070575317507	0.2204989418072976	T	T	T
0.0232100229237288	0.6334969212751145	0.2211491525635199	T	T	T
0.3562603166890955	0.6339913141480953	0.2213959205781182	T	T	T
0.6896910373013793	0.6340309989786285	0.2213788663045324	T	T	T
0.1335755840372012	0.0754435788724605	0.3194633476287911	T	T	T
0.4658490396786079	0.0755057604897510	0.3193302639088270	T	T	T
0.7993239947419004	0.0761248723715596	0.3181213836190628	T	T	T
0.1328642958801386	0.4094374597151204	0.3201006608035134	T	T	T
0.4660497524104241	0.4088929242849582	0.3185214420712605	T	T	T
0.7998939299607272	0.4089624806658726	0.3185566892168305	T	T	T
0.1329927675737651	0.7420022711322030	0.3188186527941850	T	T	T
0.4660768834184809	0.7427827363837227	0.3192504561186759	T	T	T
0.8002006499409668	0.7420417200174259	0.3188003591660344	T	T	T
0.9098617691803661	0.1863572020158298	0.4165724983149632	T	T	T
0.2413617081980177	0.1882414807578047	0.4188442027742766	T	T	T
0.5746517918925039	0.1863397227057857	0.4165681959965932	T	T	T
0.9096516781467692	0.5184437034026096	0.4182229628157428	T	T	T
0.2427636576310167	0.5183882719429856	0.4182748141244595	T	T	T
0.5760785602390700	0.5187880828498098	0.4154299011367478	T	T	T
0.9113455522701425	0.8485460708201221	0.4166726164442539	T	T	T
0.2430629395796729	0.8551764283514595	0.4172681376409536	T	T	T
0.5730377218137027	0.8550219674339747	0.4170866111690174	T	T	T
0.0161921385893373	0.9604492166706939	0.5157808743110761	T	T	T
0.3485898427854152	0.9714380079136012	0.5216827888284482	T	T	T
0.6912940920334667	0.9603795509209090	0.5157417224950097	T	T	T
0.0064078339673755	0.3006900171047454	0.5196954183802293	T	T	T
0.3610650440558852	0.3010712113190376	0.5199173332662670	T	T	T
0.6844181157622732	0.2993936998882984	0.5158182986299795	T	T	T
0.0188683353744711	0.6299341366198774	0.5218826673414257	T	T	T
0.3530066610442010	0.6385461048306298	0.5157330221229638	T	T	T
0.6763772835806754	0.6384858398523785	0.5157150961566314	T	T	T
0.8395146822196600	0.4695683082570775	0.6220789753843103	T	T	T
0.3508204728780940	0.9655128189218853	0.6344633987554946	T	T	T
0.0771352603942044	0.5105986634975663	0.6074356571429278	T	T	T
0.2809407558022748	0.4002112565609514	0.6090043370071562	T	T	T
0.3809713898559651	0.2014417934734795	0.6089059003754598	T	T	T
0.2884279708527792	0.0900613421197604	0.6073244244338383	T	T	T
0.0845460696807828	0.2005502049931713	0.6087416422353074	T	T	T
0.9847361361891532	0.3992903216311798	0.6088953142484919	T	T	T
0.0141850291296008	0.6355206448708007	0.6344128484162466	T	T	T
0.3548033029265241	0.4713746893485882	0.6223551410561577	T	T	T
0.5258555946027367	0.1312331850635386	0.6224112503937798	T	T	T
0.0104129972122613	0.1295052340347886	0.6219603404041454	T	T	T

Supplementary Table 1: Adsorption energies computed with PBC (blue), adsorption energies calculated with the finite size clusters (green), reference adsorption energies (orange), adsorption energies corrected with the additive scheme (black), errors of the corrected adsorption energies with respect to the experimental data (red).

Reaction	PW91-PBC	PBE-PBC	RPBE-PBC	SCAN-PBC	M06-Cluster	PW91-cluster	PBE-cluster	RPBE-cluster	SCAN-Cluster	Ref.	CORRECT-PW91	CORRECT-PBE	CORRECT-RPBE	CORRECT-SCAN	ERROR-PW91	ERROR-PBE	ERROR-RPBE	ERROR-SCAN
N/Ni100	-141.3	-146.7	-131.5	-149.0	-82.2	-115.3	-115.2	-102.4	-109.1	-100.4	-108.2	-113.7	-111.3	-122.1	-7.8	-13.3	-10.9	-21.6
CH/Pt111	-156.0	-153.9	-140.9	-177.3	-182.7	-173.4	-174.1	-164.1	-167.8	-163.1	-165.3	-162.5	-159.5	-192.3	-2.2	0.6	3.6	-29.2
CH_3/Pt111	-47.5	-47.1	-38.5	-64.2	-59.6	-57.0	-55.9	-49.0	-58.6	-50.9	-50.1	-50.8	-49.1	-65.2	0.8	0.1	1.8	-14.3
CO/Ni(111)	-42.5	-44.5	-33.6	-43.6	-15.7	-33.9	-33.3	-23.6	-26.1	-29.8	-24.3	-26.9	-25.7	-33.2	5.5	2.9	4.0	-3.4
CO/Pt(111)	-37.4	-38.9	-32.7	-44.9	-41.0	-49.2	-49.0	-41.3	-51.7	-29.8	-29.1	-30.9	-32.4	-34.2	0.6	-1.1	-2.7	-4.4
CO/Pd(111)	-44.9	-46.8	-36.6	-50.5	-28.8	-44.9	-44.6	-35.3	-46.1	-34.4	-28.8	-31.0	-30.1	-33.2	5.5	3.4	4.3	1.2
CO/Pd(100)	-43.3	-44.0	-36.9	-48.3	-28.6	-39.6	-39.2	-30.9	-38.7	-37.6	-32.2	-33.4	-34.6	-38.1	5.4	4.2	3.0	-0.5
CO/Rh(111)	-44.2	-43.7	-38.9	-47.0	-33.5	-43.0	-42.5	-35.7	-36.9	-33.9	-34.7	-34.8	-36.7	-43.7	-0.8	-0.8	-2.8	-9.7
CO/Ir(111)	-45.4	-45.5	-40.4	-47.0	-42.3	-47.8	-47.4	-40.6	-44.2	-39.2	-39.9	-40.4	-42.1	-45.1	-0.6	-1.2	-2.9	-5.9
CO/Cu(111)	-17.9	-17.3	-13.4	-21.0	2.0	-6.5	-5.8	1.0	-6.5	-13.6	-9.5	-9.5	-12.4	-12.5	4.1	4.1	1.2	1.1
CO/Ru(0001)	-44.0	-44.4	-38.7	-45.0	-28.8	-45.5	-45.1	-38.9	-40.3	-38.5	-27.3	-28.1	-28.7	-33.6	11.2	10.4	9.9	4.9
CO/Co(0001)	-38.7	-38.3	-32.7	-39.7	-17.2	-32.7	-31.9	-24.0	-36.1	-28.4	-23.2	-23.6	-25.9	-20.8	5.2	4.7	2.5	7.6
NO/Pt(111)	-42.3	-40.6	-35.3	-44.0	-12.7	-30.0	-29.5	-19.6	-27.6	-28.4	-24.9	-23.8	-28.4	-29.0	3.4	4.6	0.0	-0.7
NO/Pd(111)	-52.0	-54.0	-45.0	-54.8	-30.3	-44.9	-44.2	-33.5	-45.5	-43.6	-37.3	-40.0	-41.8	-39.5	6.3	3.6	1.8	4.1
NO/Pd(100)	-48.8	-51.0	-41.2	-50.5	-24.3	-39.6	-39.0	-28.3	-41.6	-39.0	-33.5	-36.3	-37.2	-33.2	5.5	2.7	1.8	5.7
O/Ni(111)	-125.9	-124.7	-113.5	-120.7	-90.7	-103.6	-102.9	-91.4	-111.4	-118.3	-113.0	-112.5	-112.8	-100.0	5.4	5.9	5.5	18.3
O/Ni(100)	-132.3	-132.5	-119.5	-131.7	-99.4	-116.1	-115.6	-103.4	-116.1	-123.6	-115.7	-116.4	-115.6	-115.1	8.0	7.3	8.0	8.6
O/Pt(111)	-97.7	-98.4	-85.7	-94.1	-48.5	-64.3	-64.3	-53.2	-56.8	-85.4	-81.9	-82.6	-80.9	-85.8	3.5	2.8	4.5	-0.4
O/Rh(100)	-121.9	-122.4	-109.1	-117.2	-65.9	-93.0	-92.3	-80.8	-85.2	-102.9	-94.8	-96.0	-94.2	-97.9	8.1	6.8	8.7	5.0
H/Pt(111)	-63.8	-62.2	-60.6	-67.5	-55.1	-58.0	-57.8	-54.3	-56.9	-63.4	-60.9	-59.6	-61.4	-65.7	2.5	3.9	2.0	-2.2
H/Ni(111)	-65.3	-65.0	-62.1	-69.5	-54.8	-56.9	-56.4	-52.6	-54.4	-66.7	-63.2	-63.3	-64.3	-69.9	3.5	3.4	2.4	-3.1
H/Ni(100)	-64.6	-64.0	-60.8	-69.6	-60.1	-59.8	-59.8	-55.5	-59.5	-65.1	-64.9	-64.3	-65.4	-70.3	0.2	0.8	-0.3	-5.2

H/Rh(111)	-65.9	-64.3	-62.8	-68.8	-56.3	-62.9	-62.9	-60.2	-61.7	-63.4	-59.3	-57.8	-58.9	-63.5	4.1	5.6	4.5	-0.1
H/Pd(111)	-66.3	-65.0	-63.2	-71.4	-60.7	-63.2	-62.9	-59.5	-63.8	-65.5	-63.8	-62.8	-64.4	-68.3	1.7	2.7	1.1	-2.8
I/Pt(111)	-57.9	-56.0	-49.9	-58.2	-49.6	-49.8	-49.0	-38.5	-48.7	-55.4	-57.6	-56.6	-61.0	-59.0	-2.3	-1.2	-5.6	-3.7
NH_3/Cu(100)	-10.0	-10.4	-8.6	-13.4	-4.9	-4.9	-4.0	1.7	-9.5	-14.3	-10.0	-11.3	-15.2	-8.8	4.3	3.0	-0.9	5.5
CH_3I/Pt(111)	-6.2	-5.4	-3.8	-9.0	-10.8	-8.9	-8.1	-0.8	-11.3	-20.1	-8.1	-8.2	-13.8	-8.5	11.9	11.9	6.3	11.6
CH_3OH/Pt(111)	-4.7	-4.5	-3.8	-16.8	-14.9	-8.4	-7.6	-1.1	-13.6	-13.1	-11.2	-11.8	-17.6	-18.1	1.9	1.4	-4.5	-4.9
CH_4/Pt(111)	-0.9	-1.0	-0.4	-2.0	-2.8	0.6	1.0	3.5	-0.7	-3.5	-4.3	-4.8	-6.7	-4.0	-0.9	-1.3	-3.2	-0.6
C_2H_6/Pt(111)	-1.2	-0.7	-0.8	-3.2	-2.9	-0.4	0.2	5.1	-2.5	-6.5	-3.6	-3.7	-8.8	-3.6	2.9	2.7	-2.3	2.9
C_3H_8/Pt(111)	-1.3	-1.1	-0.8	-4.5	-6.9	-1.6	-0.8	6.0	-4.1	-9.2	-6.7	-7.2	-13.8	-7.3	2.6	2.0	-4.6	1.9
C_4H_{10}/Pt(111)	0.1	1.0	0.8	-3.9	-13.2	-4.5	-3.5	6.0	-10.0	-9.2	-8.7	-8.7	-18.5	-7.1	0.6	0.5	-9.2	2.1
C_6H_6/Pt(111)	-21.8	-22.8	-2.7	-45.0	-36.9	-37.6	-37.6	-18.1	-49.1	-38.8	-21.0	-22.1	-21.4	-32.7	17.7	16.7	17.3	6.0
C_6H_6/Cu(111)	-6.4	-1.1	-4.7	-6.8	-9.9	-4.4	-3.5	6.1	-11.3	-15.7	-11.9	-7.5	-20.7	-5.4	3.8	8.2	-5.0	10.3
C_6H_6/Ag(111)	-1.4	-0.6	2.5	-6.1	-8.4	-1.5	-0.6	7.7	-8.8	-14.5	-8.3	-8.5	-13.7	-5.8	6.2	6.1	0.8	8.7
C_6H_6/Au(111)	4.0	-1.0	5.2	-8.9	-12.2	-6.3	-5.4	3.3	-14.2	-16.8	-1.9	-7.8	-10.3	-6.9	14.9	9.0	6.6	10.0
H_2O/Pt(111)	-5.0	-4.7	-1.1	-15.2	-10.0	-10.9	-10.3	-5.2	-13.9	-13.1	-4.2	-4.5	-5.9	-11.4	9.0	8.7	7.2	1.8
C_6H_{10}/Pt111	-17.7	-22.5	-5.5	-31.8	-30.4	-30.1	-29.4	-15.1	-42.0	-29.4	-18.0	-23.5	-20.8	-20.3	11.4	5.9	8.6	9.2

Supplementary Note 2.

Experimental uncertainties:

Experimental uncertainties were obtained as described below. Further, these values were averaged to compare to MAEs obtained theoretically.

CO/Ni(111):

Ref. (1), estimated from Figure 9 of the reference.

Ref. (2), estimated from Figure 8 of the reference.

Ref. (3), explicitly reported for a coverage close to 0. We assume a similar value for ¼ ML coverage.

CO/Pt(111):

Ref (1), estimated from Figure 11 of the reference.

Ref(4), explicitly reported for a coverage close to 0 ML – We assumed to be similar for ¼ ML coverage.

CO/Pd(111):

Ref(5), estimated from Figure 4 of the reference.

CO/Pd(100):

Ref.(6), estimated from Figure 11.

Ref.(7), explicitly shown in the reference text.

CO/Rh(111):

Ref.(8), explicitly shown in Table 1 of the reference. Since, two methods are employed, we used the average of them.

Ref. (9), Explicitly shown in the reference text for low coverage of CO on Rh(111). We, hence, assumed a similar error.

CO/Cu(111):

Ref. (10), estimated from Figure 2 in the reference.

CO/Co(0001):

Ref.(11), explicitly shown in the reference's text.

NO/Pd(111):

Ref.(12), estimated from Figure 2 of the reference by considering low NO exposure.

O/Ni(100):

Ref. (1), estimated from Figure 18 in the reference. Low coverage was assumed and the error was divided by 2 since, in the reference, the adsorption is given per O2.

O/Pt(111):

Ref. (13), Explicitly showed in Tab. 1 of the Ref.

I/Pt(111):

Ref. (14), Extracted from Fig. 5 of the reference.

NH_3/Cu(100):

Ref. (15), Explicitly given in the reference's text.

CH_3I/Pt(111):

Ref.(16), Explicitly showed in the reference for low coverages. We assume a similar uncertainty.

CH_3OH/Pt(111):

Ref. (17), Explicitly showed in the reference text for low coverages. We assumed a similar error for 0.25 ML (used coverage for our calculations).

C_6H_6/Pt(111):

Ref. (18), Computed as 1.4% of the measured data (this percentage is estimated in the reference text).

C_6H_6/Cu(111):

Ref. (19), Uncertainty estimated from Figure 8 of the reference.

D_2O/Pt(111):

Ref. (20), Explicitly written in the reference text.

CH_3/Pt111:

Ref. (21), Explicitly written in the reference text.

Supplementary Table 2: MAE and RMSE calculated using RPBE, BEEF-vdW and PBE+D3/M06. All energies in kcal mol^{-1}. Here, MAEs and RMSEs are computed by removing the reactions involving water adsorption, C_6H_6 adsorption on Pt(111), CH adsorption on Pt(111), CH_3 adsorption on (Pt111) and C_6H_{10} adsorption on Pt(111).

	RPBE[a]	BEEF-vdW[a]	PBE+D3/M06[b]
MAE-Chem.	4.2	3.7	2.2
MAE-Phys.	10.9	5.4	2.5
MAE-Tot.	6.2	4.2	2.3
RMSE-Chem.	7.4	5.9	2.5
RMSE-Phys.	12.2	6.8	3.4
RMSE-Tot.	9.1	6.2	2.8

[a] From ref. [4]
[b] Present work

Supplementary Table 3: Experimental transition barriers, barrier heights using PBE+D3 and PBC (PBE+D3-PBC), corrected barriers with the M06 hybrid (PBE+D3/M06), barriers on the finite-size cluster using PBE+D3 and M06(PBE+D3-cluster, M06-cluster), multiplicities found as the energy ground state for the cluster calculations.

Reaction	EXP.	PBE+D3-PBC	PBE+D3/M06	PBE-D3 cluster	M06 Cluster	Mult. PBE GS	Mult. PBE TS
**H_2 + Cu(111) → H_2/Cu(111)**	14.53	3.52	15.07	21.37	32.88	3	3
**H_2 + Cu(100) →H_2/Cu(100)**	17.07	6.88	17.24	8.10	18.44	1	1
**H_2 + Pt(111) → H_2/Pt(111)**	0.00	0.00	0.00	-6.64	-8.77	13	13
**CH_4 + Ni(100) →CH_4/ Ni(100)**	17.53	5.78	20.54	12.65	27.37	11	11
**CH_4 + Ni(111) →CH_4/ Ni(111)**	23.29	9.43	25.11	25.07	40.71	9	9

Supplementary Figure 1: Finite-size clusters used in our investigation.

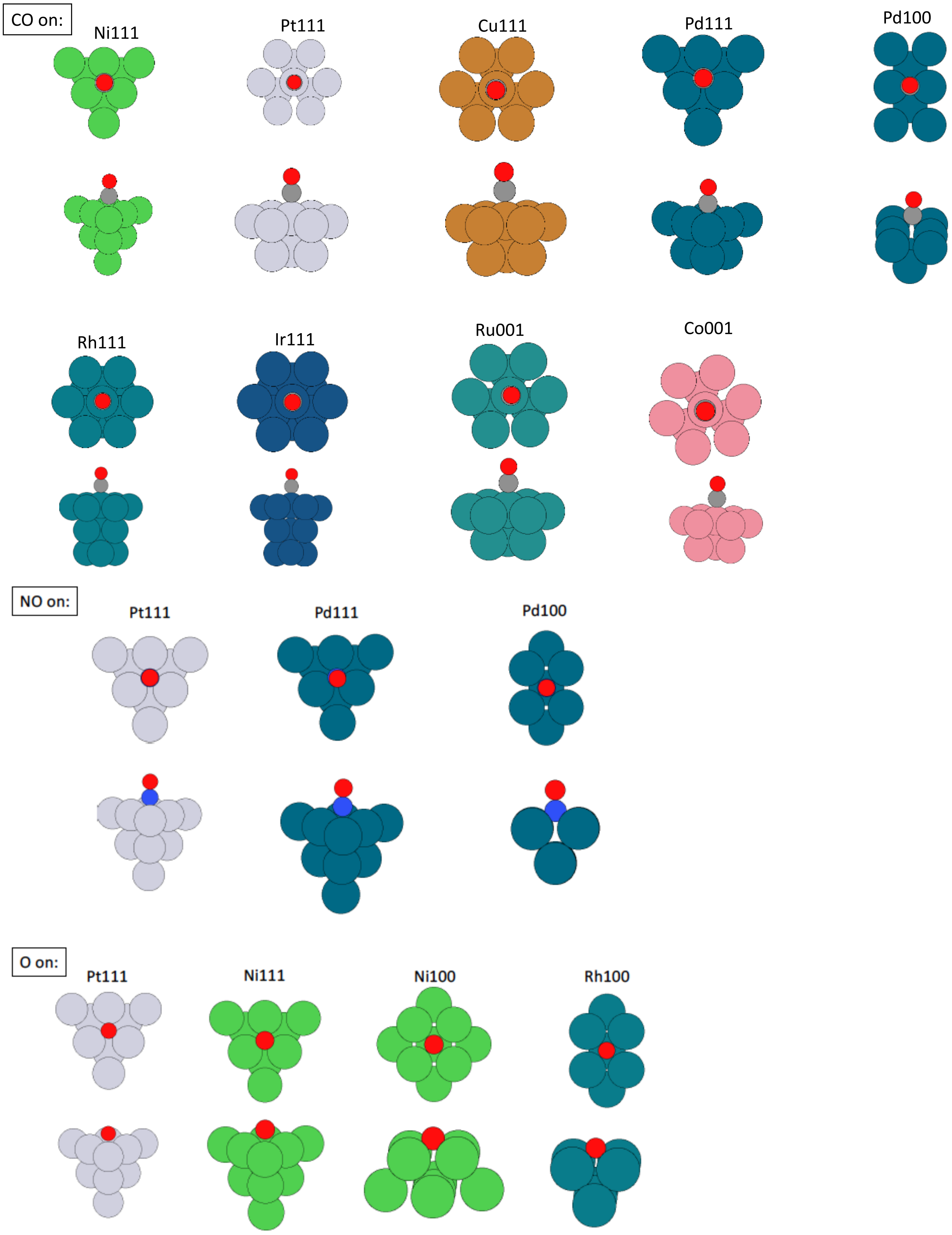

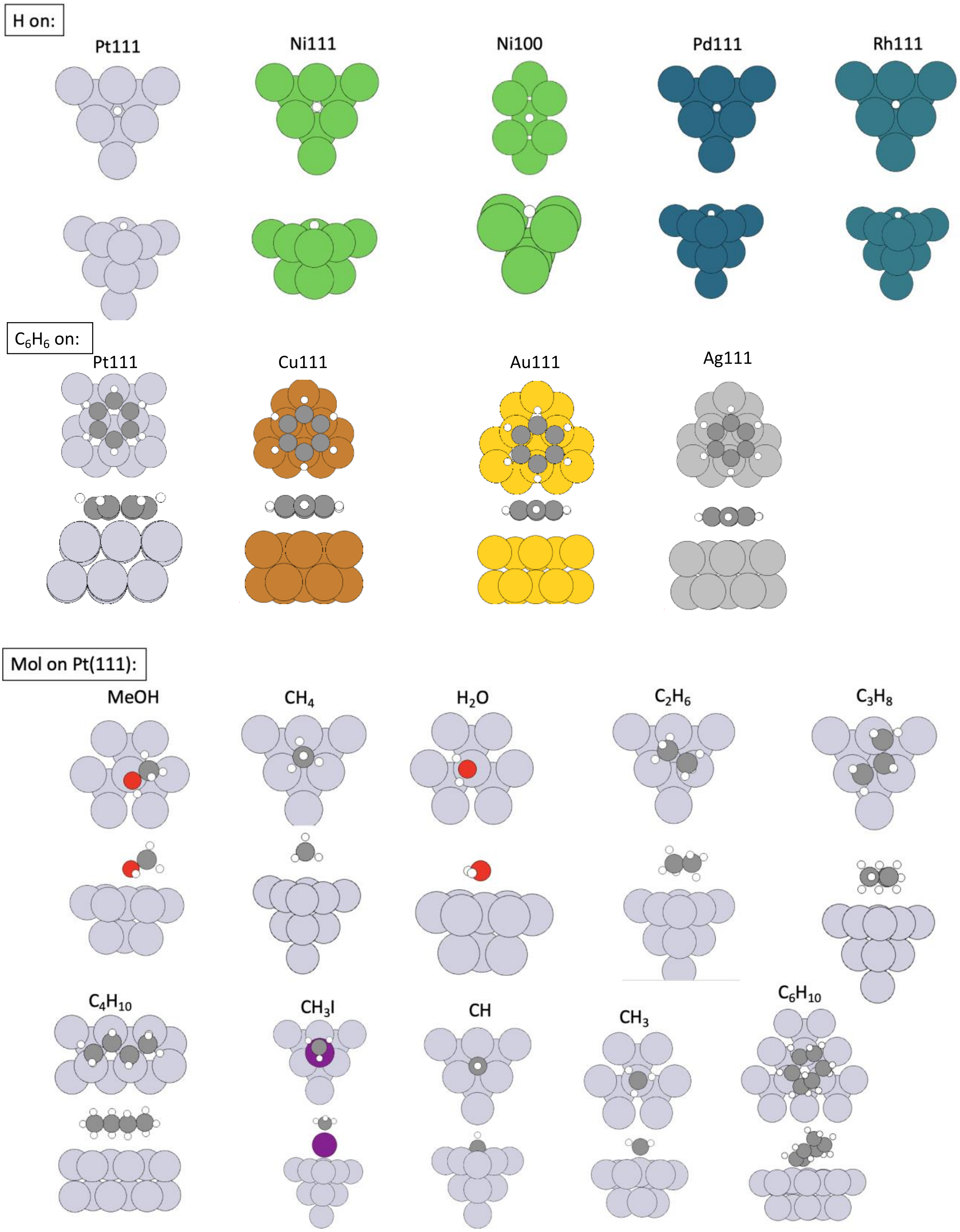
H on:
Pt111
Ni111
Ni100
Pd111
Rh111
C_6H_6 on:
Pt111
Cu111
Au111
Ag111
Mol on Pt(111):
MeOH
CH_4
H_2O
C_2H_6
C_3H_8
C_4H_{10}
CH_3I
CH
CH_3
C_6H_{10}

Other cases:

NH_3_Cu100 I_Pt111 N_Ni100

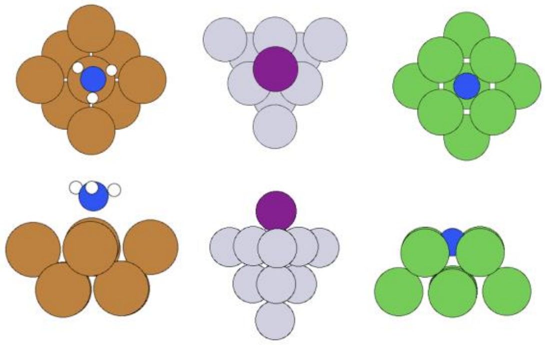

Transition States:

H_2-Pt(111)

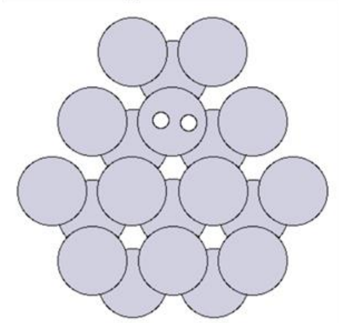

H_2-Cu(100)

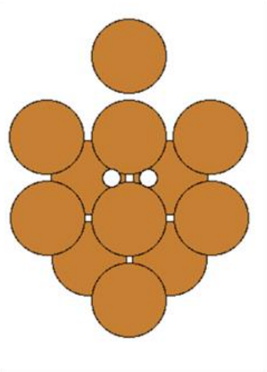

H_2-Cu(111)

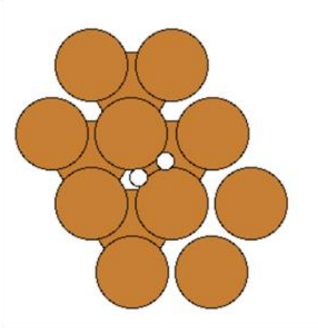

CH_4-Ni(100)

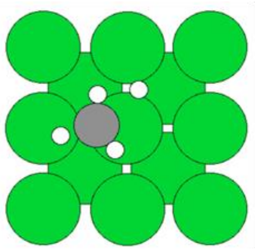

CH_4-Ni(111)

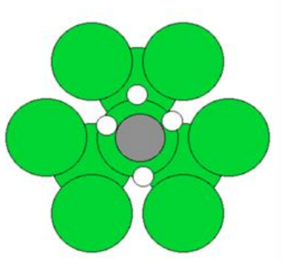

Supplementary Table 4: Optimized lattice parameters (Å). Optimization of bulk structures were performed using a *k*-point mesh of 15x15x15 for the FCC structures and 15x15x13 for the HCP structures together with a cutoff energy of 500 eV.

**Metal**	**PBE+D3**		**RPBE**	
	**a (Å)**	**c (Å)**	**a (Å)**	**c (Å)**
**Ni**	3.48		3.55	
**Pt**	3.93		3.99	
**Pd**	3.89		3.98	
**Rh**	3.79		3.85	
**Ir**	3.84		3.89	
**Cu**	3.57		3.67	
**Ag**	4.07		4.21	
**Au**	4.10		4.20	
**Co**	2.46	3.98	2.51	4.07
**Ru**	2.70	4.24	2.73	4.28

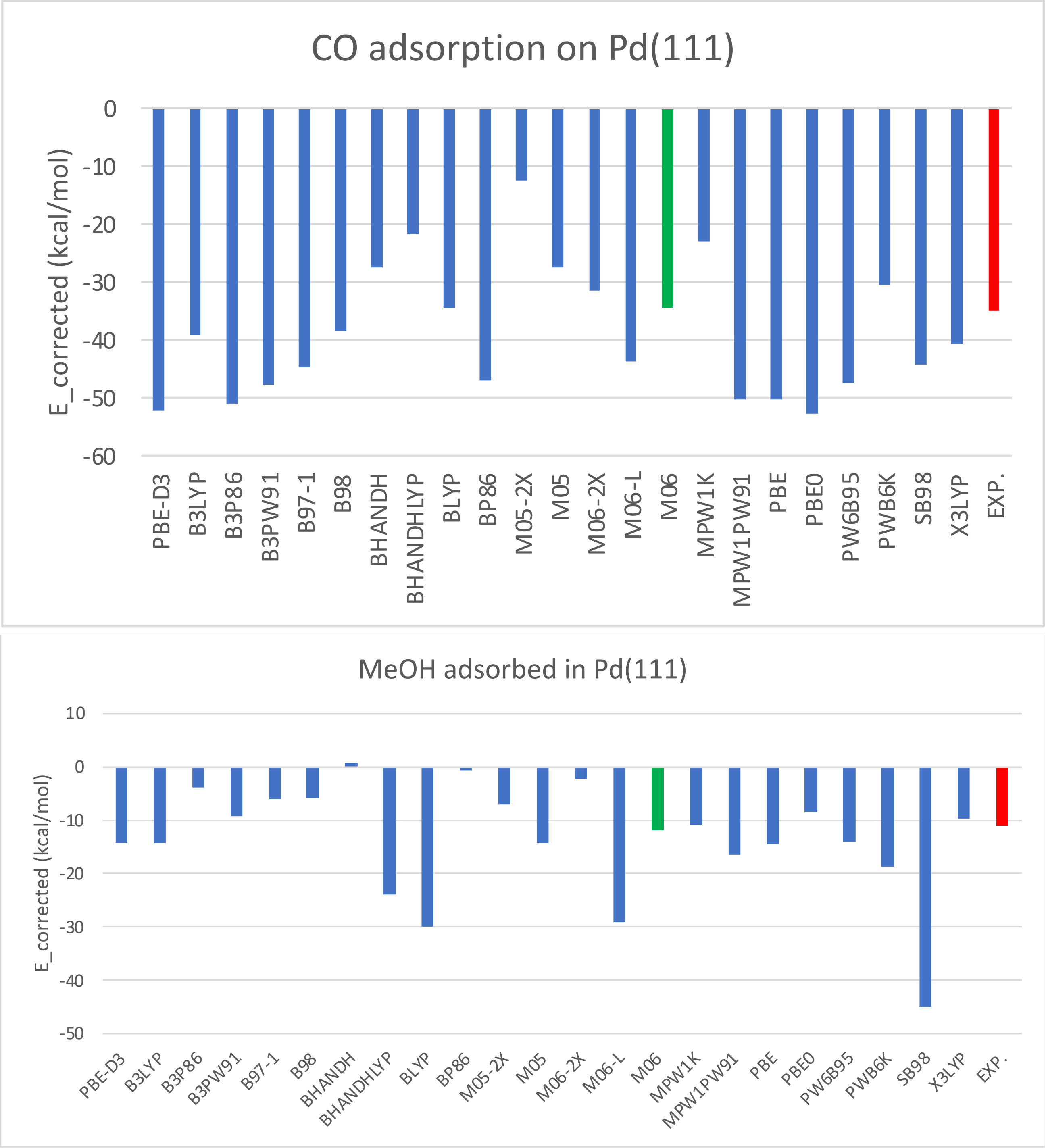

Supplementary Figure 2: (Top) Corrected CO adsorption energy and (bottom) corrected MeOH adsorption energy. Adsorption energies were calculated as $\boldsymbol{E_{ads} = E^{Ads,PBC}_{PBE+D3} + E^{Ads,Cluster}_{X} - E^{Ads,Cluster}_{PBE+D3}}$. Here, $\boldsymbol{E_{ads}}$ is the corrected adsorption energy, $\boldsymbol{E^{PBC}_{PBE+D3}}$ is the adsorption energy calculated with the PBC and using PBE+D3, $\boldsymbol{E^{Cluster}_{X}}$ is the adsorption energy calculated with the finite-size cluster with the distinct functionals (X here assumes the functionals shown in the figure), $\boldsymbol{E^{Cluster}_{PBE+D3}}$ is the finite-size adsorption energy computed using PBD+D3. Source data are provided as Source Data file.

Supplementary Table 5: Basis set superposition error, in kcal mol^{-1}, for randomly selected reactions.

Reaction	PBE+D3-BSSE	M06-BSSE	BSSE(PBE+D3-M06)
C_6H_6/Pt(111)	-5.54	-6.00	0.46
C_6H_6/Cu(111)	-2.08	-2.08	0.00
C_6H_6/Ag(111)	-1.22	-1.31	0.09
C_6H_6/Au(111)	-3.46	-3.23	-0.23
NO/Pt111	-1.38	-0.92	-0.46
CO/Pt(111)	-2.08	-1.85	-0.23
CO/Cu(111)	-1.61	-1.38	-0.23
CO/Ni(111)	-1.15	-0.46	-0.69
CO/Ru(0001)	-1.85	-1.85	0.00
O/Pt(111)	-1.15	-0.92	-0.23

Supplementary Table 6: Experimental adsorption energies (EXP.) from which zero-point energies were removed, PBE+D3 level adsorption energies using PBC (PBE+D3-PBC), adsorption energies for the finite size cluster using PBE+D3 and M06(PBE+D3-cluster, M06-cluster), corrected adsorption energies with the M06 hybrid (PBE+D3/M06), multiplicities found as the energy ground state for the cluster calculations (Mult. PBE+D3 cluster, Mult. PBE+D3 cluster-mol), and the position of the adsorbates (site).

Reaction	Exp.	PBE+D3-PBC	PBE+D3 cluster	M06 cluster	PBE+D3/M06	error	MULT. PBE+D3 cluster	MULT. PBE+D3 clus-ter+mol	Site
N/Ni100	-100.4	-146.7	-124.5	-82.2	-104.4	-3.9	9	4	fcc
CH/Pt111	-163.2	-173.7	-174.6	-166.3	-165.4	-2.2	9	6	fcc
CH_3/Pt111	-50.9	-55.0	-61.7	-59.6	-52.9	-2.0	7	6	top
CO/Ni(111)	-29.8	-49.4	-37.1	-15.7	-28.0	1.6	9	7	fcc
CO/Pt(111)	-29.8	-42.9	-54.7	-41.0	-29.3	0.2	5	5	top
CO/Pd(111)	-34.4	-51.2	-49.1	-28.8	-30.9	3.5	7	7	fcc
CO/Pd(100)	-37.6	-50.3	-43.7	-28.6	-35.1	2.5	5	3	br
CO/Rh(111)	-33.9	-49.5	-47.2	-33.5	-35.8	-1.8	20	18	top
CO/Ir(111)	-39.2	-51.2	-52.4	-42.3	-41.1	-2.1	22	20	top
CO/Cu(111)	-13.6	-23.0	-10.1	2.0	-10.9	2.8	3	3	top
CO/Ru(0001)	-38.5	-50.8	-45.9	-28.9	-33.7	4.8	21	21	top
CO/Co(0001)	-28.4	-46.6	-36.2	-17.2	-27.6	0.7	21	21	top
NO/Pt(111)	-28.4	-48.1	-34.3	-12.7	-26.6	1.8	9	8	fcc
NO/Pd(111)	-43.6	-58.1	-42.9	-30.3	-45.5	-2.1	9	8	fcc
NO/Pd(100)	-39.0	-56.1	-43.4	-24.3	-37.0	2.1	5	4	hl
O/Ni(111)	-118.3	-134.3	-104.7	-90.7	-120.2	-1.6	9	11	fcc
O/Ni(100)	-123.6	-140.7	-115.8	-99.5	-124.4	-0.7	9	9	hl
O/Pt(111)	-85.4	-106.0	-65.5	-48.5	-89.0	-3.7	9	7	fcc
O/Rh(100)	-102.9	-132.2	-94.1	-65.9	-104.0	-1.2	12	12	hl
H/Pt(111)	-63.4	-64.8	-59.5	-55.1	-60.4	3.0	9	8	top
H/Ni(111)	-66.7	-67.1	-57.9	-54.8	-64.0	2.5	9	8	fcc
H/Ni(100)	-65.1	-66.5	-61.1	-60.1	-65.6	-0.5	9	8	hl
H/Rh(111)	-63.4	-66.4	-64.1	-56.4	-58.7	4.7	15	16	fcc
H/Pd(111)	-65.5	-67.6	-64.6	-60.7	-63.7	1.8	9	8	fcc
I/Pt(111)	-55.4	-69.0	-62.7	-49.6	-55.8	-0.2	9	8	fcc
NH_3/Cu(100)	-14.3	-15.7	-7.8	-4.9	-12.7	1.6	2	2	top
CH_3I/Pt(111)	-20.1	-18.5	-16.8	-10.8	-12.5	7.4	13	13	top
CH_3OH/Pt(111)	-13.1	-14.1	-14.1	-14.9	-14.9	-1.8	7	5	top
CH_4/Pt(111)	-3.5	-6.0	-2.8	-2.8	-6.1	-2.5	9	9	fcc
C_2H_6/Pt(111)	-6.5	-10.4	-6.1	-2.9	-7.2	-0.7	9	9	fcc
C_3H_8/Pt(111)	-9.2	-15.0	-9.2	-6.9	-12.7	-3.5	9	9	top
C_4H_{10}/Pt(111)	-9.2	-20.1	-19.1	-13.2	-14.1	-4.8	15	15	top
C_6H_6/Pt(111)	-38.8	-52.4	-56.1	-36.9	-33.2	4.8	11	9	br
C_6H_6/Cu(111)	-15.7	-22.4	-16.4	-9.9	-15.9	0.0	2	2	fcc
C_6H_6/Ag(111)	-14.5	-19.4	-12.9	-8.4	-14.9	-0.5	2	2	fcc
C_6H_6/Au(111)	-16.8	-20.5	-18.2	-12.2	-14.5	2.3	2	2	fcc
H_2O/Pt(111)	-13.1	-16.8	-14.5	-10.0	-12.3	0.7	5	5	surface
C_6H_{10}/Pt111	-29.4	-43.7	-48.6	-30.4	-25.5	3.9	7	7	surface

Supplementary Note 3

Linear regression fits were performed to obtain the correlation between the reference data and the calculated adsorption energies with PBE+D3 and PBE+D3/M06 (Supplementary Figure 3). This shows that the R^2 (goodness of the linear fitting) for the PBE+D3/M06 is 0.99 confirming the good correlation between the computed and reference data, while for PBE+D3 the value is 0.96 – still correlating with the reference data, but not as well as the PBE+D3/M06. Interestingly, the different slopes revealed that, for strongly bonded adsorbates, the correction acts more intensively resulting in larger shifts while, for lower adsorption energies, smaller shifts are obtained. This confirms that our correction acts differently depending on the nature of the adsorbate interaction with the metal surface.

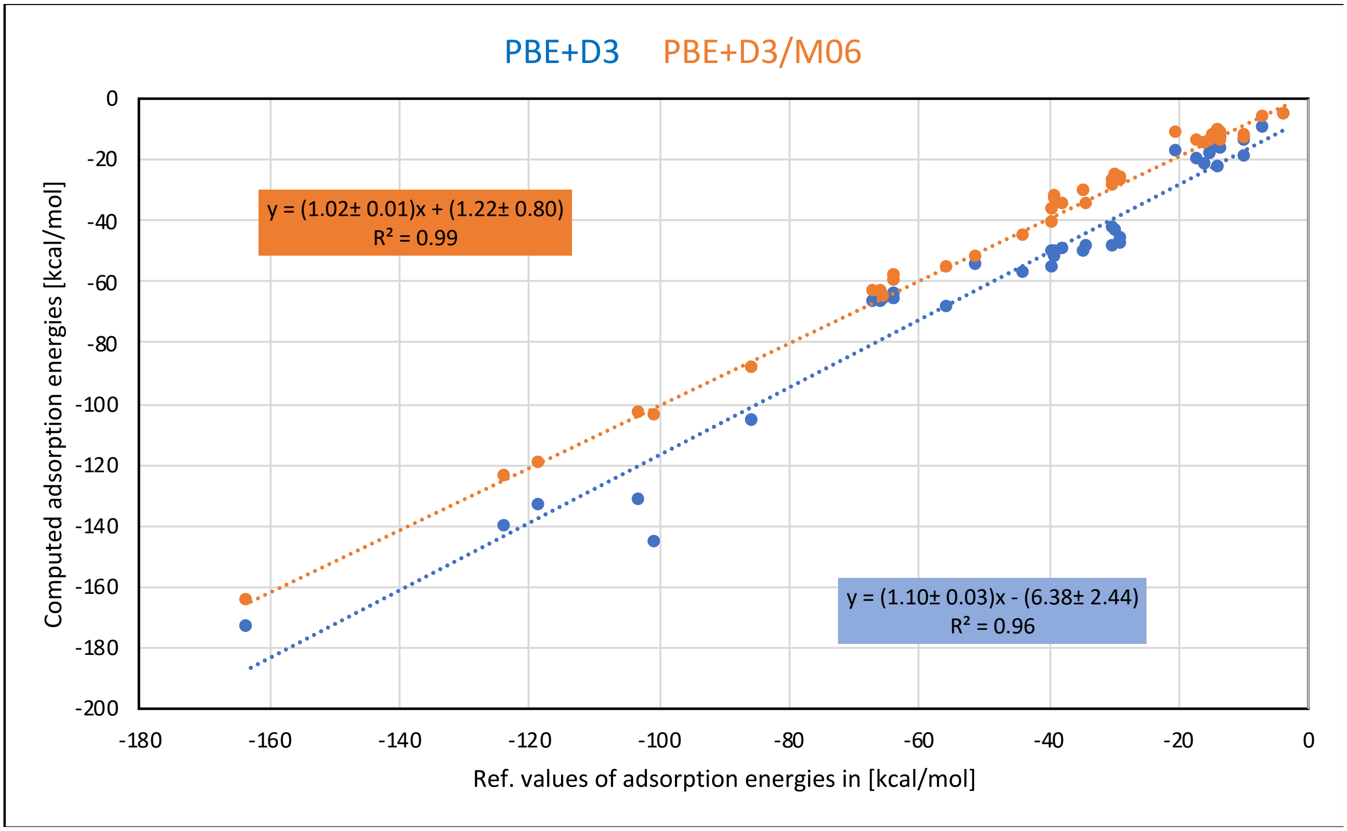

Supplementary Figure 3: Linear regression showing the correlation between the reference adsorption energies (reference data) and the values obtained with PBE+D3 (blue) and PBE+D3/M06 (orange). Source data are provided as Source Data file.

Supplementary Note 4

Structure of the employed clusters as shown in Supplementary Figure 1 (.xyz format).

C_2H_6_Pt111:
18
cluster

Pt	12.48374422	7.21008906	9.40168272
Pt	12.49993470	8.82268617	7.11773511
Pt	13.87952243	9.62688996	9.40942089
Pt	11.10174691	9.62095761	9.41450854
H	13.13636140	8.78995529	13.56234874
H	13.62627147	9.27225568	11.92590218
H	12.65549532	7.80901752	12.15918331
Pt	13.89105403	6.41483707	7.12188548
Pt	15.27321383	7.21003084	9.41988152
Pt	13.88138145	4.80437257	9.41904383
H	15.06617264	7.17812915	11.73038696
H	14.62259853	6.71423538	13.38059569
Pt	13.89464825	8.02207891	4.86898656
Pt	15.27938379	8.82100378	7.12374618
Pt	16.66262602	9.62230255	9.41825043
C	14.75362317	7.58234899	12.72061575
C	13.47868854	8.40103596	12.59236036
H	15.59605340	8.18052106	13.09329159

C_3H_8_Pt111:
21
cluster

Pt	12.50675418	8.83676071	7.12212079
Pt	12.50249780	7.24759233	9.41691996
Pt	13.89518358	9.66460530	9.40985748
Pt	11.11533582	9.65839887	9.41961470
H	13.88053287	9.72139346	11.70377568
H	13.87343398	9.82682733	13.47334775
H	12.54401229	7.53926789	13.53979375
H	12.60812748	7.44677168	11.76923648
Pt	13.89689515	6.43128998	7.12430550
Pt	13.89927219	4.84049869	9.41781672
C	13.16979791	7.20278147	12.69935544
H	13.26050362	6.10927471	12.74034562
Pt	13.89464825	8.02207891	4.86898656
Pt	15.28631266	8.83718597	7.12250834
Pt	15.29687189	7.24588078	9.41588481
Pt	16.68322183	9.65883645	9.41276805
C	14.52736420	7.89112325	12.69064977
C	14.43345693	9.40902981	12.62236995
H	15.11115534	7.58915271	13.57623640
H	15.11145642	7.52725625	11.81182438
H	15.42102002	9.88862261	12.57680371

C_4H_{10}_Pt111:
38
cluster

Pt	11.10454414	6.42514596	7.11620693
Pt	11.08550981	4.83370194	9.42111551
Pt	12.49392185	8.83276341	7.12265210
Pt	9.71348407	8.83183940	7.12028316
Pt	13.88316107	11.24006669	7.11990071
Pt	11.10401429	11.23968730	7.12294001
Pt	12.47140808	7.23100697	9.41560737
Pt	9.69832257	7.23667674	9.41258680
Pt	13.86562727	9.65768124	9.41417028
Pt	11.08418790	9.64561869	9.41483391
Pt	15.25390428	12.05357296	9.42111551
Pt	12.47402606	12.05079368	9.42208109
C	12.63848768	8.11824103	12.67376479
H	11.76387034	8.78060171	12.61446892
H	12.57958712	7.43000987	11.80181950
H	12.54821394	7.49704845	13.57784499
Pt	13.88274375	6.42518817	7.12117327
Pt	16.66035789	6.42547780	7.12095664
Pt	13.86454332	4.82749244	9.42329988
Pt	16.64242053	4.83092266	9.42208109
Pt	15.27049529	8.83191127	7.12615124
Pt	18.05027302	8.83183940	7.12028316
Pt	16.66163856	11.23911515	7.12243772
Pt	15.25504021	7.22770248	9.41719349
Pt	18.03511152	7.23667674	9.41258680
Pt	16.65027583	9.65059592	9.41663491
Pt	19.42097685	9.64561869	9.41483391
Pt	18.03293780	12.04736346	9.42329988
C	13.94535277	8.89726966	12.65118042
C	15.19201342	8.01943512	12.64918859
C	16.49860917	8.79626617	12.72612378
H	13.98510837	9.60411691	13.49823820
H	13.96999653	9.54366229	11.73664516
H	15.18846649	7.40860015	11.70876087
H	15.13449815	7.28098454	13.46756528
H	17.37353056	8.13292939	12.67169018
H	16.58099676	9.50823737	11.87729405
H	16.56405179	9.38742984	13.65280629

C_6H_6_Ag111:
25
cluster

Ag	10.08369770	4.15323033	7.49500286
Ag	11.52322801	6.64662692	7.49529121
Ag	11.51771702	4.98801299	9.84693778
Ag	11.52270380	1.66004148	7.49403569
Ag	14.40205642	1.65970685	7.49535511
Ag	12.96231410	4.15317799	7.50393501

Ag	15.84231110	4.15312758	7.49560784
Ag	14.40237726	6.64672181	7.49426410
Ag	10.07805634	2.48806951	9.85476295
Ag	12.96318889	2.47669526	9.84593819
Ag	15.84977187	2.48650678	9.85102400
Ag	14.41605474	4.99168482	9.84482284
Ag	12.96267391	7.48457247	9.85303696
H	10.93646741	5.34926916	12.86156350
C	13.09460183	5.50320147	12.85437275
C	14.30806487	4.80430160	12.84268792
C	14.30718870	3.40387927	12.84319099
C	13.09504825	2.70260004	12.84702887
C	11.88275322	3.40361527	12.85826649
C	11.88163033	4.80379409	12.86052728
H	13.09469020	6.59458072	12.83670268
H	15.25290114	5.34967778	12.82864271
H	15.25256056	2.85843452	12.81812214
H	13.09568991	1.61151290	12.83629374
H	10.93800118	2.85747000	12.84387869

C_6H_6_Au111:
25
cluster

Au	10.15002349	4.17605360	7.22742009
Au	11.60189771	6.68807151	7.22711592
Au	11.59876483	1.66369356	7.22677150
Au	14.50162416	1.66331528	7.22642591
Au	13.05157885	4.17579112	7.23982056
Au	15.95278619	4.17699734	7.22745659
Au	14.50196770	6.68930091	7.22717102
Au	10.14834627	2.49609626	9.63185471
Au	13.05264932	2.47636359	9.61060587
Au	15.95690461	2.49506927	9.62845443
Au	11.59336641	5.01613038	9.61674029
Au	14.51669277	5.01661747	9.61366417
Au	13.05504342	7.52529803	9.63402218
H	10.95759287	5.32187206	12.67974921
C	13.11614728	5.47469702	12.68354355
C	14.32944466	4.77535504	12.66912079
C	14.32776483	3.37490834	12.66526565
C	13.11539405	2.67397109	12.65755341
C	11.90375150	3.37601953	12.67266284
C	11.90281645	4.77615056	12.67798468
H	13.11662210	6.56501619	12.67314729
H	15.27470864	5.32019848	12.66446752
H	15.27234954	2.82902323	12.64103500
H	13.11502324	1.58302807	12.64396562
H	10.95859995	2.83055610	12.65417498

C_6H_6_Cu111:
25
cluster

Cu	7.57170004	1.45859568	6.51615833
Cu	5.05071188	1.45924472	6.51504041
Cu	8.83343916	3.64339317	6.51495284
Cu	3.78997912	3.64325290	6.51526395
Cu	6.31193215	3.64306545	6.54605923
Cu	5.04994454	5.82644466	6.51621937
Cu	7.57260587	5.82696626	6.51481551
Cu	3.78009739	2.18093212	8.56844093
Cu	6.31169573	2.16657485	8.58411055
Cu	5.03362679	4.38051312	8.58458057
Cu	7.59150689	4.38148417	8.58277098
Cu	6.31180445	6.56578696	8.56890291
Cu	8.84344405	2.18088894	8.56868246
C	6.34097246	5.05077191	11.34898468
C	7.55605855	4.35065495	11.34499064
C	7.55373195	2.94787789	11.34794377
C	6.33949206	2.24616142	11.34870489
C	5.12653167	2.94961373	11.35184390
C	5.12564335	4.35162924	11.35061437
H	6.34188375	6.14240468	11.34084805
H	8.50123622	4.89501926	11.34388177
H	8.49884794	2.40117943	11.34097872
H	6.33877276	1.15567972	11.35152148
H	4.18137725	2.40428052	11.34744582
H	4.18143533	4.89737290	11.35387275

C_6H_6_Pt111:
27
cluster

Pt	12.53169669	8.81951902	7.11736263
Pt	13.90533434	11.21480254	7.15698980
Pt	12.48884767	12.07730979	9.38406973
Pt	13.92183100	6.42921244	7.13021967
Pt	16.66276914	6.43025807	7.12996328
Pt	15.29281937	8.83729397	7.16332612
Pt	18.05564930	8.82036605	7.11689501
Pt	16.68271269	11.21636789	7.15290701
Pt	12.49213394	7.18169737	9.38061056
Pt	15.30951171	7.25914956	9.52679642
Pt	18.12099113	7.17993712	9.37831653
Pt	13.82455157	9.64465876	9.49357908
Pt	16.79304075	9.64644165	9.48568560
Pt	15.30698514	12.02064527	9.55368308
Pt	18.12432374	12.08135360	9.38560696
H	13.17074983	10.84643478	11.87005820
H	15.32794246	7.20056959	12.12139794
C	15.31901108	11.13320334	11.51893416
C	16.55831547	10.33835534	11.54882127
C	16.56119858	8.90430019	11.53999538
C	15.32344827	8.10676746	11.50564540
C	14.08364606	8.90041487	11.54715233
C	14.08173859	10.33502836	11.55424410
H	15.32062262	12.03354610	12.14382602
H	17.47152519	10.85162781	11.85587481
H	17.47658637	8.39113017	11.84201429
H	13.17309622	8.38345289	11.85643229

CH_3I_Pt111:
22
cluster

Pt	6.92943330	3.98772978	6.93367869
Pt	8.30107510	4.77650432	9.24106745
Pt	5.55712647	4.77606254	9.24039051
Pt	8.31854403	6.39058298	6.92550886
Pt	6.92900115	7.18724905	9.21506713
H	8.72158844	6.64818489	14.22688174
Pt	11.09040261	1.58958309	6.92550886
Pt	8.31077133	1.58937460	6.92662553
Pt	12.47315046	3.98772978	6.93367869
Pt	9.70067832	3.99658750	6.92905732
Pt	13.84479226	4.77650432	9.24106745
Pt	11.10084363	4.77606254	9.24039051
Pt	6.92928725	2.39629815	9.25967388
Pt	9.70085973	2.38624915	9.21506713
Pt	11.08262991	6.39037450	6.92662553
Pt	9.70114584	7.19729805	9.25967388
Pt	12.47271831	7.18724905	9.21506713
C	9.65247138	6.10514913	14.04569704
H	9.67965454	5.14778657	14.57357626
H	10.53376135	6.71617590	14.25643118
I	9.70684792	5.62444055	11.86205804
Pt	12.47300442	2.39629815	9.25967388

CH_3OH_Pt111:
16
cluster

Pt	13.85302543	6.41808348	6.92799076
Pt	11.08786518	6.42077998	6.92077573
Pt	12.46678239	8.81134677	6.92967118
Pt	11.07485987	4.84643030	9.22286048
Pt	9.69221556	7.24812983	9.21457620
Pt	12.45612902	7.26181514	9.25140666
C	13.74080144	7.51401265	12.35636623
O	12.64587929	6.83523472	11.71054465
H	14.67006434	7.43318779	11.76130892
H	12.96069016	5.95014803	11.40730899
H	13.45979412	8.57107454	12.42813410
H	13.90242613	7.09489905	13.36213521
Pt	11.07456760	9.64406247	9.23906159
Pt	13.84671845	9.64743019	9.22286048
Pt	13.84642618	4.84306258	9.23906159
Pt	15.23593273	7.24812983	9.21457620

CH_4_Pt111:
15
cluster
Pt 8.31127085 6.40147512 6.92844491
Pt 8.30321046 4.80038015 9.23609008
Pt 6.91952762 7.20286231 9.24022630
H 9.42053360 6.74876582 12.09240950
Pt 9.69730440 4.00118742 6.93001033
Pt 9.69138620 2.40186242 9.24022630
Pt 9.70150504 5.60116655 4.66321306
Pt 11.08307500 6.40204326 6.92913631
Pt 11.07739895 4.80267430 9.23664994
Pt 9.68991772 7.20507304 9.23459744
Pt 12.46324479 7.20286231 9.24022630
C 9.66600104 5.77045809 12.52900428
H 10.65664586 5.45446640 12.17835727
H 8.92001932 5.03528360 12.20253234
H 9.66563086 5.84752411 13.62347571

CO_Co001:
12
cluster
Co 4.92377685 5.69177440 27.11318984
Co 6.14913510 3.55109016 27.12549101
Co 8.62530043 3.55120927 27.12523221
Co 9.84977685 5.69177440 27.11318984
Co 7.38695601 5.68432721 27.02177856
Co 6.14913510 7.81713130 27.12549101
Co 8.62530043 7.81725041 27.12523221
Co 7.38726701 4.26873810 29.07003288
Co 6.15790452 6.39338626 29.05118476
Co 8.61676347 6.39324225 29.05101031
C 7.38117516 5.78275254 25.28760726
O 7.37808508 5.86255939 24.12289398

CO_Cu111:
12
cluster
Cu 5.03462013 1.46349251 6.21948262
Cu 2.53464325 1.46316604 6.21791562
Cu 3.78496205 3.62771905 6.22029783
Cu 2.52406054 -0.00321329 8.24806447
Cu 1.25957416 2.18806106 8.24776863
Cu 3.78439153 2.18483059 8.36015032
Cu 2.52200660 4.37059927 8.24818176
Cu 5.04843175 4.36912590 8.24806447
Cu 5.04637781 -0.00173992 8.24818176
Cu 6.30831658 2.18806106 8.24776863
C 3.74376599 2.13942999 10.20225189
O 3.72068414 2.11228427 11.35951369

CO_Ir111:
15
cluster
Ir 10.85471934 9.39994915 8.68535774
Ir 13.57000938 4.69692885 8.68535774
Ir 12.21880518 5.48685702 4.31702503
Ir 10.86116016 7.83836718 4.31702503
Ir 13.57645020 7.83836718 4.31702503
Ir 13.57122118 6.27356238 6.51405183
Ir 10.86651278 6.27343213 6.51398772
Ir 12.21875752 8.61583216 6.51379716
Ir 10.86805026 4.69700147 8.68517231
Ir 9.50404140 7.06275498 8.68567736
Ir 12.21925725 7.05230723 8.90590832
Ir 13.58334030 9.40002177 8.68517231
Ir 14.93462148 7.06275498 8.68567736
C 12.22143130 7.03387929 10.75490411
O 12.22357123 7.01710872 11.91847315
CO_Ni111:
12
cluster
Ni 9.83726957 2.83977511 3.60802424
Ni 9.84197817 1.42312592 5.60705587
Ni 11.07142539 3.55082962 5.60420218
Ni 8.61445027 3.55020900 5.60744709

Ni 8.61967684 2.13331427 7.67724373
Ni 7.39165638 4.26428660 7.58945527
Ni 9.85060301 4.26802295 7.66821531
Ni 9.85097378 0.00462391 7.58945527
Ni 11.08419999 2.13169863 7.66506220
Ni 12.31029118 4.26428660 7.58945527
C 9.86637993 2.84982308 8.99711331
O 9.88945322 2.85708680 10.18684731

CO_Pd100:
10
cluster
Pd 6.87294445 6.87760142 6.29147945
Pd 6.87336215 9.63279604 6.29235930
Pd 5.49637774 5.51021257 8.26173682
Pd 8.24681335 5.51007879 8.26171582
Pd 5.48588536 8.26038737 8.32514484
Pd 8.23838790 8.26144452 8.33485139
Pd 5.49637774 11.01150332 8.26173682
Pd 8.24681335 11.01136955 8.26171582
C 6.85618505 8.33845818 9.75869963
O 6.85024942 8.39496113 10.93493394

CO_Pd111:
11
cluster
Pd 8.25324455 6.35227522 6.78811119
Pd 9.62818241 8.73366836 6.78817587
Pd 6.88030468 8.73258693 6.78882030
Pd 5.50535103 9.52613962 9.04189958
Pd 6.87336986 7.13969314 9.12556848
Pd 8.25615161 9.53509085 9.12546511
Pd 8.25599641 4.76188207 9.04189958
Pd 11.00664179 9.52613962 9.04189958
Pd 9.63876994 7.13975023 9.12185650
C 8.26076412 7.93578949 10.43894557
O 8.26376835 7.93427988 11.62760827

CO_Pt111:
12
cluster
Pt 5.52889351 1.60928810 6.94006791
Pt 2.78718046 1.60921297 6.94054149
Pt 4.15808040 3.98343901 6.94020997
Pt 2.76992817 0.00168932 9.19080887
Pt 1.38626726 2.39824377 9.19084668
Pt 4.15765076 2.40084959 9.39868752
Pt 2.77424284 4.80267617 9.19081793
Pt 5.54178675 4.80268922 9.19080887
Pt 5.54610142 0.00167627 9.19081793
Pt 6.92998442 2.39824377 9.19084668
C 4.16682362 2.40414988 11.23747497
O 4.17306219 2.40643617 12.39570154

CO_Rh111:
15
cluster
Rh 10.68927721 9.26091401 8.80178886
Rh 12.02788635 5.40112488 4.48238402
Rh 10.69145453 7.71589269 4.48238402
Rh 13.36431816 7.71589269 4.48238402
Rh 13.36461816 6.17467136 6.65331218
Rh 10.69219042 6.17479363 6.65382476
Rh 12.02844158 8.48977906 6.65176999
Rh 13.36214084 4.63137840 8.80178886
Rh 10.69537532 4.63162328 8.80181224
Rh 9.35597484 6.94976758 8.80113945
Rh 12.02868459 6.94743341 8.99228886
Rh 13.36823895 9.26115889 8.80181224
Rh 14.70170211 6.94976758 8.80113945
C 12.03795213 6.99123423 10.81779994
O 12.04412671 7.02192630 11.98145547

CO_Ru100:
12
cluster
Ru 5.09864852 1.11152893 21.25916311

Ru 3.77468054 3.45666080 21.25862305
Ru 5.11564767 4.23468330 23.30929466
Ru 5.09864852 5.78460201 21.25916311
Ru 7.84172344 1.10922272 21.25900042
Ru 6.47277792 3.44199246 21.12616763
Ru 9.17068054 3.45666080 21.25862305
Ru 7.81994702 4.23486624 23.30923017
Ru 6.46721421 1.88052035 23.33136756
Ru 7.84172344 5.78229579 21.25900042
C 6.52063798 3.41944075 19.24483422
O 6.55697498 3.40895685 18.07838639

H_Ni100:
8
cluster
Ni 6.14622995 3.69351428 7.49751729
Ni 6.14574795 6.15337507 7.47696287
Ni 6.14622995 8.61214905 7.49751729
Ni 4.91951016 4.93188246 9.23389000
Ni 7.37234274 4.92882592 9.23444272
Ni 4.91506602 7.38600143 9.23583026
Ni 7.36828141 7.38137396 9.23494095
H 6.14010681 6.16495236 9.81808587

H_Ni111:
10
cluster
Ni 7.38381283 5.68312716 5.60721577
Ni 6.15976809 6.39828949 7.60204870
Ni 8.61244599 3.55502200 5.60660215
Ni 8.61908549 2.13862680 7.60204870
Ni 9.84238773 5.68391876 5.60798454
Ni 7.37796631 4.26169949 7.63751607
Ni 9.85676407 4.26296153 7.63823602
Ni 8.61769288 6.40825896 7.63854326
Ni 11.07840289 6.39828949 7.60204870
H 8.61525439 4.97625919 8.55271530

H_Pd111:
11
cluster
Pd 13.75614395 6.36116818 6.78913198
Pd 12.38012256 7.15829396 9.05627867
Pd 15.12975826 3.98020469 6.78801776
Pd 15.13076794 2.39403641 9.05627867
Pd 15.12854958 5.55830047 4.54589255
Pd 16.50428585 6.36143315 6.78920298
Pd 13.73884898 4.76575545 9.08466647
Pd 16.52143608 4.76632910 9.08408277
Pd 15.12978109 7.17851247 9.08456322
Pd 17.88141332 7.15829396 9.05627867
H 15.13079167 5.56818699 9.90653643

H_Pt111:
11
cluster
Pt 13.83723166 4.78142046 9.26959566
Pt 13.86454218 6.39799243 6.93137809
Pt 12.47661319 7.19685582 9.20797407
Pt 15.24585819 4.00375300 6.93020390
Pt 16.65927916 4.78231219 9.26971117
Pt 15.24847177 2.39585592 9.20797407
Pt 15.24522220 5.60116655 4.66321306
Pt 16.62834945 6.39860079 6.93002693
Pt 15.24967819 7.22689978 9.26982772
Pt 18.02033035 7.19685582 9.20797407
H 15.24959802 5.58914228 10.16425973

H_Rh111:
11
cluster
Rh 8.02016156 6.18221292 6.63805764
Rh 8.00130747 4.63626407 8.82135544
Rh 6.68814444 6.96362689 8.80677722
Rh 9.35756957 3.86508423 6.63670298
Rh 9.36100807 2.33409127 8.80677722
Rh 9.35502272 5.40112488 4.48238402

Rh 10.69484388 6.18205497 6.63839809
Rh 10.72052579 4.63439954 8.82063660
Rh 9.36109973 6.99088573 8.82157704
Rh 12.03387170 6.96362689 8.80677722
H 9.37022030 5.42279899 9.78952550

H_2O_Pt111:
13
cluster
Pt 6.96836618 4.00325525 6.92504550
Pt 8.35455752 6.40278168 6.92742865
Pt 5.63343295 4.80926117 9.22678926
Pt 7.01835158 7.20822426 9.23325817
Pt 9.73487271 4.00957904 6.92694572
Pt 7.02045853 2.40585823 9.23382403
Pt 9.79021016 2.40722437 9.23325817
Pt 8.42362846 4.80757318 9.24384518
Pt 11.17715012 4.80926117 9.22678926
Pt 9.79231712 7.20685812 9.23382403
H 7.42716613 5.47417891 11.63353300
H 7.60763196 3.93843104 11.61412808
O 8.11308383 4.77554579 11.70524621

I_Pt111:
11
cluster
Pt 13.82882845 4.78083429 9.24999405
Pt 13.86658619 6.39719385 6.90968685
Pt 12.47455061 7.20022797 9.15514429
Pt 15.24536592 4.00888326 6.90947581
Pt 16.66535313 4.78072275 9.24881578
Pt 15.24640919 2.39922808 9.15514429
Pt 15.24522220 5.60116655 4.66321306
Pt 16.62408310 6.39702165 6.90960631
Pt 15.24651659 7.23650927 9.25068382
Pt 18.01826777 7.20022797 9.15514429
I 15.25298881 5.59262132 11.42538883

NH_3_Cu001:
13
cluster
Cu 1.27481431 1.26948832 5.90147840
Cu 3.78184473 1.26929264 5.90315678
Cu 1.27495335 3.77689153 5.90152952
Cu 3.78172216 3.77714709 5.90304952
Cu 2.52592656 -0.00251336 7.64831081
Cu 0.01275388 2.52269160 7.64664940
Cu 2.52867637 2.51753308 7.78710196
Cu 2.52592656 5.04622905 7.64831081
Cu 5.06149630 2.52269160 7.64664940
N 2.64525931 2.56191655 9.89322148
H 2.59950145 1.61620159 10.28213491
H 3.54152815 2.98846596 10.14631218
H 1.88306263 3.12713926 10.27723314

NO_Pd100:
9
cluster
Pd 6.87930938 4.12807388 6.29591723
Pd 6.87783073 6.87896290 6.26348737
Pd 6.87930938 9.62936464 6.29591723
Pd 5.52057178 5.52071228 8.31802108
Pd 8.23845230 5.52275056 8.31937999
Pd 5.52031872 8.24002705 8.32049363
Pd 8.24200440 8.24396997 8.31781288
N 6.88960819 6.89241795 9.35139034
O 6.89578353 6.89879709 10.57230225

NO_Pd111:
12
cluster
Pd 13.75322689 7.94042925 4.54589255
Pd 13.75575217 6.35259579 6.78487769
Pd 15.12982622 8.73241349 6.78496489
Pd 12.38196247 8.73221889 6.78443069
Pd 11.00552835 9.52686002 9.01655156
Pd 12.36217685 7.13409567 9.12951946

Pd 13.75634108 9.54855968 9.12920803
Pd 13.75617373 4.76260247 9.01655156
Pd 16.50681911 9.52686002 9.01655156
Pd 15.15084425 7.13375759 9.12832417
N 13.75846183 7.93782068 10.36286201
O 13.76127778 7.93640337 11.57179759

NO_Pt111:
12
cluster
Pt 6.92122606 4.01231119 6.92499392
Pt 5.51141485 4.83326373 9.16179610
Pt 8.31557575 3.20066660 4.66321306
Pt 8.29974785 1.62481167 6.92248341
Pt 9.67874885 4.01300002 6.92199045
Pt 8.28327343 0.03226383 9.16179610
Pt 6.86319291 2.41116649 9.31684151
Pt 9.69924977 2.41563540 9.31588029
Pt 8.28178799 4.87178134 9.31812713
Pt 11.05513201 4.83326373 9.16179610
N 8.27628810 3.23774828 10.59269260
O 8.27554135 3.23935902 11.80539685

O_Ni100:
10
cluster
Ni 3.68635694 6.15432145 7.50126510
Ni 6.14570962 3.69468715 7.50143119
Ni 6.14559966 6.15428832 7.43625248
Ni 8.60499171 6.15432145 7.50126510
Ni 6.14570962 8.61332192 7.50143119
Ni 4.91520357 4.92397300 9.27075557
Ni 7.37561222 4.92454653 9.27171733
Ni 4.91493119 7.38426800 9.27181113
Ni 7.37550822 7.38498715 9.27038584
O 6.14576246 6.15316226 10.13339731

O_Ni111:
11
cluster
Ni 9.84329618 9.94219077 5.60810200
Ni 8.62037690 10.65920749 7.57337492
Ni 11.07161552 7.81470721 5.60806949
Ni 11.07969430 6.39954480 7.57337492
Ni 11.06692829 9.22926915 3.60802424
Ni 12.29909396 9.94148211 5.60600184
Ni 9.82440451 8.51466353 7.68408161
Ni 12.33382255 8.51523074 7.68245588
Ni 11.07956852 10.68837147 7.68223456
Ni 13.53901170 10.65920749 7.57337492
O 11.07961087 9.23926167 8.80878205

O_Pt111:
11
cluster
Pt 5.55785930 3.20883156 4.86898656
Pt 5.55773842 1.61048720 7.12957538
Pt 4.17355948 4.00787271 7.12960104
Pt 6.94180111 4.00781988 7.12965687
Pt 5.55755744 -0.01697148 9.38884839
Pt 4.07389980 2.35190524 9.47139205
Pt 7.04128388 2.35205806 9.47138893
Pt 2.76414575 4.82120748 9.38897237
Pt 5.55755115 4.92166311 9.47149813
Pt 8.35121013 4.82144587 9.38917186
O 5.55776541 3.20866965 10.58066404

O_Rh111:
8
cluster
Rh 6.72034778 4.02942595 6.76566505
Rh 6.72077898 6.71673819 6.73636120
Rh 6.72034778 9.40343749 6.76566505
Rh 5.38562284 5.37932355 8.66490009
Rh 8.06067256 5.37709096 8.66441574
Rh 5.38401322 8.05330926 8.66405018
Rh 8.05768778 8.05136581 8.66517382

O 6.72476541 6.71230279 9.64723375

N_Ni100
10
test

Ni	3.68753990	6.15183821	7.48049009
Ni	6.14644624	3.69102148	7.47997127
Ni	6.14764684	6.14918701	7.47265805
Ni	8.60617467	6.15183821	7.48049009
Ni	6.14644624	8.60965625	7.47997127
Ni	4.89209096	4.90039446	9.29676122
Ni	7.39903514	4.89968286	9.29693297
Ni	4.89316450	7.40574091	9.29815936
Ni	7.39917108	7.40541580	9.29715400
N	6.14613417	6.15223449	9.72555914

CH_Pt111
12
test

Pt	8.30567751	6.38003108	6.94063881
Pt	6.90580301	7.17087491	9.19921236
Pt	9.68732388	3.98868906	6.94136990
Pt	9.67766160	2.36987502	9.19921236
Pt	9.70150504	5.60116655	4.66321306
Pt	11.06986902	6.38053734	6.93946860
Pt	8.25643292	4.75060901	9.34345527
Pt	11.09576450	4.75127494	9.33671040
Pt	9.67852234	7.20475662	9.33174283
Pt	12.44952018	7.17087491	9.19921236
C	9.67749754	5.56867464	10.47939396
H	9.67517322	5.56412195	11.57631774

C_6H_{10}_Pt111
34
test

Pt	11.10558976	4.83159364	9.36694215
Pt	16.69143632	1.60208552	7.10022384
Pt	11.14554627	1.61615586	7.15665292
Pt	13.88764459	1.61881253	7.14608011
Pt	12.51583019	4.01026265	7.13814680
Pt	15.27889626	4.01937867	7.13905812
Pt	13.90969962	6.39803416	7.15092643
Pt	11.13960759	0.00592376	9.41973859
Pt	13.91257037	0.00168698	9.40258102
Pt	9.75013020	2.40663612	9.41332560
Pt	12.53086540	2.44196363	9.59102062
Pt	15.33907969	2.39257577	9.37804288
Pt	13.90282082	4.79346308	9.55639170
Pt	16.69249752	4.82056855	9.40006539
C	14.53796877	2.35333356	13.45512216
C	15.49586701	3.39919647	12.89056905
C	13.60323257	1.85936806	12.34438626
C	14.72119512	4.64190184	12.46927875
C	12.86923458	2.99250046	11.62709498
C	13.49706154	4.35759167	11.59621811
H	15.08535959	1.49475763	13.87250940
H	13.95279129	2.79532401	14.28153641
H	16.26860861	3.67704365	13.62318160
H	16.02693600	2.97639361	12.01507255
H	14.23207411	1.29594075	11.62701076
H	12.86267106	1.13540148	12.71401521
H	14.34561879	5.15369046	13.37617110
H	15.38135260	5.37067836	11.97366304
H	11.81530990	3.06514819	11.93465177
H	12.72585580	5.12710323	11.74339726
Pt	12.53132154	7.22237252	9.40369878
Pt	15.30800206	7.22579478	9.41973859
Pt	16.69971601	0.00250150	9.40369878
Pt	18.08691915	2.40663612	9.41332560

CH_3_Pt111
14
cluster

Pt	5.53715368	1.61168495	6.94155256
Pt	2.78688683	1.61083524	6.94208469

```
Pt    4.16200503    3.99329205    6.94149763
Pt    2.78012750    0.01615641    9.20355317
Pt    1.39645192    2.41028638    9.20427125
Pt    4.16901071    2.41655047    9.40431102
Pt    2.78235422    4.81569174    9.20400762
Pt    5.55198608    4.81715631    9.20355317
Pt    5.55421281    0.01469185    9.20400762
Pt    6.94016908    2.41028638    9.20427125
C     4.20317611    2.46065314    11.47087154
H     5.22221945    2.71294650    11.78965043
H     3.49147495    3.22498207    11.80653368
H     3.91391124    1.46639300    11.82871089
```

Pt111 (2x2x1 supercell)

```
Pt
   1.00000000000000
     5.5437171645025325    0.0000000000000000    0.0000000000000000
     2.7718585822512662    4.8009998958550284    0.0000000000000000
     0.0000000000000000    0.0000000000000000   26.7896391656699997
   Pt
    16
Selective dynamics
Direct
  0.0000000000000000  0.0000000000000000  0.0895868729383835   F   F   F
  0.5000000000000000  0.0000000000000000  0.0895868729383835   F   F   F
  0.0000000000000005  0.5000000000000002  0.0895868729383835   F   F   F
  0.4999999999999999  0.5000000000000002  0.0895868729383835   F   F   F
  0.1666666666666641  0.1666666666666648  0.1740677814428793   F   F   F
  0.6666666666666649  0.1666666666666648  0.1740677814428793   F   F   F
  0.1666666666666637  0.6666666666666662  0.1740677814428793   F   F   F
  0.6666666666666641  0.6666666666666662  0.1740677814428793   F   F   F
  0.8333340069926380  0.3333338726708500  0.2585297093494143   T   T   T
  0.3333326223805705  0.3333332933412504  0.2585299340394671   T   T   T
  0.8333333434590441  0.8333326644556263  0.2585300433509711   T   T   T
  0.3333336527823206  0.8333337629046895  0.2585295821347396   T   T   T
 -0.0000000210450377 -0.0000000499522746  0.3445157136434697   T   T   T
  0.5000002940609619 -0.0000000445545241  0.3445158048506652   T   T   T
  0.0000000286842452  0.5000002714609068  0.3445160339986025   T   T   T
  0.5000000121744990  0.5000000965673168  0.3445156525588364   T   T   T
```

Pt111 (3x3x1 supercell)

```
Pt
   1.00000000000000
     8.3367889501893959    0.0000000000000000    0.0000000000000000
     4.1683944750946980    7.2198710168534168    0.0000000000000000
     0.0000000000000000    0.0000000000000000   26.8069596737456877
   Pt
    36
Selective dynamics
Direct
  0.0000000000000000  0.0000000000000000  0.0969897381740878   F   F   F
  0.3333333333333357  0.0000000000000000  0.0969897381740878   F   F   F
  0.6666666666666643  0.0000000000000000  0.0969897381740878   F   F   F
```

```
 0.0000000000000000  0.3333333333333357  0.0969897381740878   F   F   F
 0.3333333333333357  0.3333333333333357  0.0969897381740878   F   F   F
 0.6666666666666643  0.3333333333333357  0.0969897381740878   F   F   F
 0.0000000000000000  0.6666666666666643  0.0969897381740878   F   F   F
 0.3333333333333357  0.6666666666666643  0.0969897381740878   F   F   F
 0.6666666666666643  0.6666666666666643  0.0969897381740878   F   F   F
 0.1111111111111143  0.1111111111111143  0.1816314351636024   F   F   F
 0.4444444444444429  0.1111111111111143  0.1816314351636024   F   F   F
 0.7777777777777786  0.1111111111111143  0.1816314351636024   F   F   F
 0.1111111111111143  0.4444444444444429  0.1816314351636024   F   F   F
 0.4444444444444429  0.4444444444444429  0.1816314351636024   F   F   F
 0.7777777777777786  0.4444444444444429  0.1816314351636024   F   F   F
 0.1111111111111143  0.7777777777777786  0.1816314351636024   F   F   F
 0.4444444444444429  0.7777777777777786  0.1816314351636024   F   F   F
 0.7777777777777786  0.7777777777777786  0.1816314351636024   F   F   F
 0.8888890127519341  0.2222222269445771  0.2657372577890565   T   T   T
 0.2222218816651465  0.2222228417991479  0.2657370201733298   T   T   T
 0.5555552861251661  0.2222225573689326  0.2657371559939429   T   T   T
 0.8888894457456541  0.5555554038043884  0.2657371609688066   T   T   T
 0.2222220487673936  0.5555557135098972  0.2657371083946404   T   T   T
 0.5555551988293859  0.5555555182767979  0.2657373123685480   T   T   T
 0.8888893700380062  0.8888884505426345  0.2657372228834760   T   T   T
 0.2222220117660632  0.8888884817776871  0.2657372482952844   T   T   T
 0.5555557475453665  0.8888888133851119  0.2657371999500746   T   T   T
 0.0000004759649399 -0.0000005228963283  0.3512615566675056   T   T   T
 0.3333327064827917 -0.0000000683193930  0.3512614395061254   T   T   T
 0.6666672125309349 -0.0000001814741417  0.3512617915718652   T   T   T
-0.0000000750959787  0.3333336970037389  0.3512610855082949   T   T   T
 0.3333326310824495  0.3333344680702237  0.3512612761610956   T   T   T
 0.6666665215500053  0.3333337238109174  0.3512618121721231   T   T   T
 0.0000007206509594  0.6666662864920799  0.3512610734764204   T   T   T
 0.3333327762314443  0.6666665690862045  0.3512609140923444   T   T   T
 0.6666670715322063  0.6666660735684334  0.3512613764164751   T   T   T
```

Rh(100) (2x2x1 supercell)

```
Rh
  1.00000000000000
    5.3740115370177612    0.0000000000000000    0.0000000000000000
    0.0000000000000000    5.3740115370177612    0.0000000000000000
    0.0000000000000000    0.0000000000000000   25.6999999999999993
  Rh
   16
Selective dynamics
Direct
 0.2500000000000000  0.2500000000000000  0.1167315175097272   F   F   F
 0.7500000000000000  0.2500000000000000  0.1167315175097272   F   F   F
 0.2500000000000000  0.7500000000000000  0.1167315175097272   F   F   F
 0.7500000000000000  0.7500000000000000  0.1167315175097272   F   F   F
 0.0000000000000000  0.0000000000000000  0.1906614785992247   F   F   F
 0.5000000000000000  0.0000000000000000  0.1906614785992247   F   F   F
```

```
 0.0000000000000000  0.5000000000000000  0.1906614785992247   F   F   F
 0.5000000000000000  0.5000000000000000  0.1906614785992247   F   F   F
 0.2499970670278453  0.2500042972389692  0.2642456701054445   T   T   T
 0.7500029323735170  0.2500042746375212  0.2642454338421469   T   T   T
 0.2499970801520135  0.7499956939426405  0.2642457853936055   T   T   T
 0.7500029187124853  0.7499957334065528  0.2642459399056453   T   T   T
-0.0000001247046301  0.0000012938291801  0.3356567255106531   T   T   T
 0.5000000758375223  0.0000012441933546  0.3356567449585329   T   T   T
-0.0000000648602555  0.4999987535321702  0.3356594289893680   T   T   T
 0.5000001129703053  0.4999987136853782  0.3356573371091065   T   T   T
```

Rh(111) (2x2x1 supercell)

```
Rh
   1.00000000000000
     5.3457272657702992    0.0000000000000000    0.0000000000000000
     2.6728636328851496    4.6295356138602060    0.0000000000000000
     0.0000000000000000    0.0000000000000000   26.5471520526103575
   Rh
    16
Selective dynamics
Direct
 0.0000000000000000  0.0000000000000000  0.0866382953411318   F   F   F
 0.5000000000000001  0.0000000000000000  0.0866382953411318   F   F   F
 0.0000000000000002  0.5000000000000003  0.0866382953411318   F   F   F
 0.5000000000000003  0.5000000000000003  0.0866382953411318   F   F   F
 0.1666666666666645  0.1666666666666646  0.1688461349320534   F   F   F
 0.6666666666666647  0.1666666666666646  0.1688461349320534   F   F   F
 0.1666666666666642  0.6666666666666654  0.1688461349320534   F   F   F
 0.6666666666666653  0.6666666666666654  0.1688461349320534   F   F   F
 0.8333230516337994  0.3333406107762537  0.2499703275640218   T   T   T
 0.3333313242273475  0.3333389215226080  0.2499704467606713   T   T   T
 0.8333295381365132  0.8333328268765028  0.2499673554897362   T   T   T
 0.3333339084307911  0.8333284533198703  0.2499675610309310   T   T   T
-0.0000514267889501  0.0000185858453438  0.3317004551080254   T   T   T
 0.5000563533788362 -0.0000142453039552  0.3317018464391515   T   T   T
-0.0000085658086618  0.5000025189806266  0.3316817654853931   T   T   T
 0.5000565074991359  0.4999706982994521  0.3316862514267272   T   T   T
```

Pd111 (2x2x1 supercell)

```
Pd
   1.00000000000000
     5.5012907576313399    0.0000000000000000    0.0000000000000000
     2.7506453788156700    4.7642575497132809    0.0000000000000000
     0.0000000000000000    0.0000000000000000   26.7376776414429358
   Pd
    16
Selective dynamics
Direct
 0.0000000000000000  0.0000000000000000  0.0860209338613274   F   F   F
 0.4999999999999999  0.0000000000000000  0.0860209338613274   F   F   F
```

0.0000000000000002 0.4999999999999992 0.0860209338613274 F F F
0.5000000000000001 0.4999999999999992 0.0860209338613274 F F F
0.1666666666666643 0.1666666666666642 0.1700182270169037 F F F
0.6666666666666642 0.1666666666666642 0.1700182270169037 F F F
0.1666666666666647 0.6666666666666633 0.1700182270169037 F F F
0.6666666666666641 0.6666666666666633 0.1700182270169037 F F F
0.8333333457958625 0.3333334756716129 0.2535242171355883 T T T
0.3333332901871255 0.3333333137939591 0.2535242599488715 T T T
0.8333332533682236 0.8333332640695104 0.2535242498715624 T T T
0.3333334662358985 0.8333333547853732 0.2535242223323442 T T T
-0.0000000076487719 -0.0000000288590316 0.3384668089787924 T T T
0.5000000232380537 0.0000000980640834 0.3384668590585695 T T T
0.0000000992940489 0.5000000110020998 0.3384668657885029 T T T
0.4999999525034253 0.4999999600183490 0.3384666735230565 T T T
Pd(100) (2x2x1 supercell)
Pd
1.00000000000000
5.5012907576313399 0.0000000000000000 0.0000000000000000
0.0000000000000000 5.5012907576313399 0.0000000000000000
0.0000000000000000 0.0000000000000000 25.8350000000000009
Pd
16
Selective dynamics
Direct
0.2500000000000000 0.2500000000000000 0.0928972324366200 F F F
0.7500000000000001 0.2500000000000000 0.0928972324366200 F F F
0.2500000000000000 0.7500000000000001 0.0928972324366200 F F F
0.7500000000000001 0.7500000000000001 0.0928972324366200 F F F
0.0000000000000000 0.0000000000000000 0.1681826978904582 F F F
0.5000000000000000 0.0000000000000000 0.1681826978904582 F F F
0.0000000000000000 0.5000000000000000 0.1681826978904582 F F F
0.5000000000000000 0.5000000000000000 0.1681826978904582 F F F
0.2500000942472548 0.2499998867895026 0.2434721742191240 T T T
0.7499998995094297 0.2499998814148580 0.2434721702878453 T T T
0.2500000918990114 0.7500001245409789 0.2434721861013840 T T T
0.7499999101231194 0.7500001172761029 0.2434721823963721 T T T
0.0000000063098411 0.0000000076093677 0.3187796361574931 T T T
0.4999999963839423 0.0000000122798470 0.3187795785730169 T T T
0.0000000084500169 0.4999999819925317 0.3187796549181975 T T T
0.4999999922514839 0.4999999896234494 0.3187796098185537 T T T

Ni111 (2x2x1 supercell)
Ni
1.00000000000000
4.9186348000000004 0.0000000000000000 0.0000000000000000
2.4593174000000002 4.2596626887381914 0.0000000000000000
0.0000000000000000 0.0000000000000000 25.0240727000000014
Ni
16

```
Selective dynamics
Direct
  0.0000000000000000  0.0000000000000000  0.0639384331711952   F   F   F
  0.4999999969436288  0.0000000000000000  0.0639384331711952   F   F   F
  0.0000000000000000  0.4999999969436288  0.0639384331711952   F   F   F
  0.4999999969436288  0.4999999969436288  0.0639384331711952   F   F   F
  0.1666666656478739  0.1666666656478739  0.1441821353181041   F   F   F
  0.6666666625915028  0.1666666656478739  0.1441821353181041   F   F   F
  0.1666666656478739  0.6666666625915028  0.1441821353181041   F   F   F
  0.6666666625915028  0.6666666625915028  0.1441821353181041   F   F   F
  0.8333333343521261  0.3333333312957478  0.2244258374650201   T   T   T
  0.3333333312957478  0.3333333312957478  0.2244258374650201   T   T   T
  0.8333333343521261  0.8333333282393767  0.2244258374650201   T   T   T
  0.3333333312957478  0.8333333282393767  0.2244258374650201   T   T   T
  0.0000000000000000  0.0000000000000000  0.3046695396119290   T   T   T
  0.4999999969436288  0.0000000000000000  0.3046695396119290   T   T   T
  0.0000000000000000  0.4999999969436288  0.3046695396119290   T   T   T
  0.4999999969436288  0.4999999969436288  0.3046695396119290   T   T   T
```

Ni100 (2x2x1 supercell)

```
Ni
   1.00000000000000
     4.9186347699336253    0.0000000000000000    0.0000000000000000
     0.0000000000000000    4.9186347699336253    0.0000000000000000
     0.0000000000000000    0.0000000000000000   25.2169999999999987
   Ni
    16
Selective dynamics
Direct
  0.2500000000000000  0.2500000000000000  0.1586231510488926   F   F   F
  0.7500000000000000  0.2500000000000000  0.1586231510488926   F   F   F
  0.2500000000000000  0.7500000000000000  0.1586231510488926   F   F   F
  0.7500000000000000  0.7500000000000000  0.1586231510488926   F   F   F
  0.0000000000000000  0.0000000000000000  0.2275845659674042   F   F   F
  0.5000000000000000  0.0000000000000000  0.2275845659674042   F   F   F
  0.0000000000000000  0.5000000000000000  0.2275845659674042   F   F   F
  0.5000000000000000  0.5000000000000000  0.2275845659674042   F   F   F
  0.2499972418774926  0.2499978387553272  0.2976164466529927   T   T   T
  0.7500027321396995  0.2499978777901851  0.2976177335537874   T   T   T
  0.2499972868433138  0.7500021934983504  0.2976181782045113   T   T   T
  0.7500027670063548  0.7500021886834187  0.2976161373281354   T   T   T
  0.0000003540775612 -0.0000000911168804  0.3656279130677312   T   T   T
  0.4999996619011077 -0.0000000942104551  0.3656248764911313   T   T   T
  0.0000003427523623  0.5000001020034130  0.3656257030276616   T   T   T
  0.4999996534699727  0.5000000966731378  0.3656230221101746   T   T   T
```

Ir111 (2x2x1 supercell)

```
Ir
```

```
  1.00000000000000
    5.4305800795126853    0.0000000000000000    0.0000000000000000
    2.7152900397563426    4.7030203061437010    0.0000000000000000
    0.0000000000000000    0.0000000000000000   26.6510751010644888
  Ir
   16
Selective dynamics
Direct
  0.0000000000000000  0.0000000000000000  0.0787960707790063   F   F   F
  0.5000000000000000  0.0000000000000000  0.0787960707790063   F   F   F
  0.0000000000000003  0.5000000000000000  0.0787960707790063   F   F   F
  0.5000000000000000  0.5000000000000000  0.0787960707790063   F   F   F
  0.1666666666666644  0.1666666666666643  0.1619831476710587   F   F   F
  0.6666666666666644  0.1666666666666643  0.1619831476710587   F   F   F
  0.1666666666666643  0.6666666666666643  0.1619831476710587   F   F   F
  0.6666666666666643  0.6666666666666643  0.1619831476710587   F   F   F
  0.8333310831596678  0.3333334536144148  0.2441236808616243   T   T   T
  0.3333336985974243  0.3333375547282915  0.2441219422996846   T   T   T
  0.8333371080254190  0.8333323390909156  0.2441212108412593   T   T   T
  0.3333323302357448  0.8333292583826618  0.2441224966304276   T   T   T
 -0.0000004286931938 -0.0000027588085721  0.3263411947726383   T   T   T
  0.5000015411667580  0.0000000260692540  0.3263430430857564   T   T   T
  0.0000006680352649  0.5000028064661819  0.3263417003060597   T   T   T
  0.4999976646082094  0.5000004285366602  0.3263403955064122   T   T   T
```

Cu111 (2x2x1 supercell)

```
Cu
  1.00000000000000
    5.0487424176719493    0.0000000000000000    0.0000000000000000
    2.5243712088359747    4.3723391908679723    0.0000000000000000
    0.0000000000000000    0.0000000000000000   26.1834213830208924
  Cu
   16
Selective dynamics
Direct
  0.0000000000000000  0.0000000000000000  0.0802034222067647   F   F   F
  0.5000000000000000  0.0000000000000000  0.0802034222067647   F   F   F
  0.0000000000000000  0.5000000000000000  0.0802034222067647   F   F   F
  0.5000000000000000  0.5000000000000000  0.0802034222067647   F   F   F
  0.1666666666666642  0.1666666666666644  0.1589227167884672   F   F   F
  0.6666666666666643  0.1666666666666644  0.1589227167884672   F   F   F
  0.1666666666666641  0.6666666666666646  0.1589227167884672   F   F   F
  0.6666666666666642  0.6666666666666646  0.1589227167884672   F   F   F
  0.8333331176655858  0.3333331209366991  0.2375655716641815   T   T   T
  0.3333331037116481  0.3333330955763999  0.2375655915542818   T   T   T
  0.8333330698173287  0.8333331268680317  0.2375655779609254   T   T   T
  0.3333331192599565  0.8333331261027411  0.2375655801916680   T   T   T
  0.0000000328022348  0.0000000936740210  0.3163401961246302   T   T   T
  0.5000000535403289  0.0000001123265679  0.3163402594893535   T   T   T
  0.0000000891453730  0.5000000346958073  0.3163401800932025   T   T   T
```

0.5000000716556622 0.5000000170874668 0.3163402064769956   T   T   T

Cu100 (3x3x1 supercell)

```
Cu
   1.00000000000000
     5.0487424176719493    0.0000000000000000    0.0000000000000000
     0.0000000000000000    5.0487424176719493    0.0000000000000000
     0.0000000000000000    0.0000000000000000   25.3550000000000004
   Cu
    16
Selective dynamics
Direct
  0.2500000000000000  0.2500000000000000  0.0907118911457303   F   F   F
  0.7500000000000000  0.2500000000000000  0.0907118911457303   F   F   F
  0.2500000000000000  0.7500000000000000  0.0907118911457303   F   F   F
  0.7500000000000000  0.7500000000000000  0.0907118911457303   F   F   F
  0.0000000000000000  0.0000000000000000  0.1611122066653508   F   F   F
  0.5000000000000000  0.0000000000000000  0.1611122066653508   F   F   F
  0.0000000000000000  0.5000000000000000  0.1611122066653508   F   F   F
  0.5000000000000000  0.5000000000000000  0.1611122066653508   F   F   F
  0.2499998935473775  0.2499995446668574  0.2325656101994300   T   T   T
  0.7500000213604063  0.2499999978659114  0.2325657184118313   T   T   T
  0.2499996404513687  0.7499998633728657  0.2325656869095045   T   T   T
  0.7500001069257126  0.7499999405162877  0.2325655613210251   T   T   T
 -0.0000002430745693 -0.0000001828257389  0.3025281032004022   T   T   T
  0.5000000209109416  0.0000001692174809  0.3025280615530543   T   T   T
  0.0000000880902709  0.4999999979772046  0.3025281546410095   T   T   T
  0.5000002769019379  0.5000002203140921  0.3025281483420720   T   T   T
```

Cu111 (3x3x1 supercell)

```
Cu
 1.0000000000000000
     7.5731136265079240    0.0000000000000000    0.0000000000000000
     3.7865568132539620    6.5585087863019584    0.0000000000000000
     0.0000000000000000    0.0000000000000000   26.1834213830208924
  36
Selective dynamics
Cartesian
  0.0000000000000000 -0.0000000000000005  2.4000000000000199   F   F   F
  2.5243712088359747 -0.0000000000000005  2.4000000000000199   F   F   F
  5.0487424176719493 -0.0000000000000005  2.4000000000000199   F   F   F
  1.2621856044179873  2.1861695954339861  2.4000000000000199   F   F   F
  3.7865568132539620  2.1861695954339861  2.4000000000000199   F   F   F
  6.3109280220899366  2.1861695954339861  2.4000000000000199   F   F   F
  2.5243712088359747  4.3723391908679723  2.4000000000000199   F   F   F
  5.0487424176719493  4.3723391908679723  2.4000000000000199   F   F   F
  7.5731136265079240  4.3723391908679723  2.4000000000000199   F   F   F
  1.2621856044179873  0.7287231984779954  4.4611404610069911   F   F   F
  3.7865568132539620  0.7287231984779954  4.4611404610069911   F   F   F
  6.3109280220899366  0.7287231984779954  4.4611404610069911   F   F   F
```

```
 2.5243712088359747  2.9148927939119815  4.4611404610069911   F   F   F
 5.0487424176719493  2.9148927939119815  4.4611404610069911   F   F   F
 7.5731136265079240  2.9148927939119815  4.4611404610069911   F   F   F
 3.7865568132539620  5.1010623893459677  4.4611404610069911   F   F   F
 6.3109280220899366  5.1010623893459677  4.4611404610069911   F   F   F
 8.8352992309259122  5.1010623893459677  4.4611404610069911   F   F   F
 0.0000000000000000  1.4574463969559908  6.5222809220139553   T   T   T
 2.5243712088359747  1.4574463969559908  6.5222809220139553   T   T   T
 5.0487424176719502  1.4574463969559908  6.5222809220139553   T   T   T
 1.2621856044179873  3.6436159923899765  6.5222809220139553   T   T   T
 3.7865568132539620  3.6436159923899765  6.5222809220139553   T   T   T
 6.3109280220899375  3.6436159923899765  6.5222809220139553   T   T   T
 2.5243712088359747  5.8297855878239631  6.5222809220139553   T   T   T
 5.0487424176719493  5.8297855878239631  6.5222809220139553   T   T   T
 7.5731136265079240  5.8297855878239631  6.5222809220139553   T   T   T
 0.0000000000000000 -0.0000000000000005  8.5834213830209176   T   T   T
 2.5243712088359747 -0.0000000000000005  8.5834213830209176   T   T   T
 5.0487424176719493 -0.0000000000000005  8.5834213830209176   T   T   T
 1.2621856044179873  2.1861695954339861  8.5834213830209176   T   T   T
 3.7865568132539620  2.1861695954339861  8.5834213830209176   T   T   T
 6.3109280220899366  2.1861695954339861  8.5834213830209176   T   T   T
 2.5243712088359747  4.3723391908679723  8.5834213830209176   T   T   T
 5.0487424176719493  4.3723391908679723  8.5834213830209176   T   T   T
 7.5731136265079240  4.3723391908679723  8.5834213830209176   T   T   T
```

Au111 (2x2x1 supercell)

```
Au
   1.00000000000000
     5.7982756057296898    0.0000000000000000    0.0000000000000000
     2.8991378028648449    5.0214539727055145    0.0000000000000000
     0.0000000000000000    0.0000000000000000   27.1014083110323973
   Au
    16
Selective dynamics
Direct
 0.0000000000000000  0.0000000000000000  0.0811765932881201   F   F   F
 0.5000000000000000  0.0000000000000000  0.0811765932881201   F   F   F
 0.0000000000000000  0.5000000000000000  0.0811765932881201   F   F   F
 0.5000000000000001  0.5000000000000000  0.0811765932881201   F   F   F
 0.1666666666666643  0.1666666666666643  0.1685202499907845   F   F   F
 0.6666666666666644  0.1666666666666643  0.1685202499907845   F   F   F
 0.1666666666666644  0.6666666666666643  0.1685202499907845   F   F   F
 0.6666666666666645  0.6666666666666643  0.1685202499907845   F   F   F
 0.8333333084065306  0.3333333125703085  0.2554606123748515   T   T   T
 0.3333333027980924  0.3333333164461311  0.2554606141130945   T   T   T
 0.8333333167520310  0.8333333009899736  0.2554606137444545   T   T   T
 0.3333333093657330  0.8333333072889002  0.2554606127756900   T   T   T
-0.0000000110373802 -0.0000000080477693  0.3442307110993361   T   T   T
 0.4999999940252081 -0.0000000095310658  0.3442307100591347   T   T   T
-0.0000000060804608  0.4999999911802346  0.3442307118656107   T   T   T
```

```
 0.4999999905662276  0.4999999905515556  0.3442307107199422   T   T   T
```

Au111 (3x3x1 supercell)

```
Au
   1.00000000000000
     8.6974134085945352    0.0000000000000000    0.0000000000000000
     4.3487067042972676    7.5321809590582713    0.0000000000000000
     0.0000000000000000    0.0000000000000000   27.1014083110323973
   Au
    36
Selective dynamics
Direct
  0.0000000000000000  0.0000000000000000  0.0922461287365056   F   F   F
  0.3333333333333360  0.0000000000000000  0.0922461287365056   F   F   F
  0.6666666666666644  0.0000000000000000  0.0922461287365056   F   F   F
  0.0000000000000008  0.3333333333333350  0.0922461287365056   F   F   F
  0.3333333333333373  0.3333333333333350  0.0922461287365056   F   F   F
  0.6666666666666663  0.3333333333333350  0.0922461287365056   F   F   F
  0.0000000000000011  0.6666666666666643  0.0922461287365056   F   F   F
  0.3333333333333358  0.6666666666666643  0.0922461287365056   F   F   F
  0.6666666666666649  0.6666666666666643  0.0922461287365056   F   F   F
  0.1111111111111148  0.1111111111111143  0.1795897854391633   F   F   F
  0.4444444444444429  0.1111111111111143  0.1795897854391633   F   F   F
  0.7777777777777793  0.1111111111111143  0.1795897854391633   F   F   F
  0.1111111111111144  0.4444444444444428  0.1795897854391633   F   F   F
  0.4444444444444434  0.4444444444444428  0.1795897854391633   F   F   F
  0.7777777777777787  0.4444444444444428  0.1795897854391633   F   F   F
  0.1111111111111150  0.7777777777777778  0.1795897854391633   F   F   F
  0.4444444444444454  0.7777777777777778  0.1795897854391633   F   F   F
  0.7777777777777806  0.7777777777777778  0.1795897854391633   F   F   F
  0.8888888752855114  0.2222222070093568  0.2667014493011255   T   T   T
  0.2222222081480872  0.2222222101755627  0.2667014498880764   T   T   T
  0.5555555442885847  0.2222222081859509  0.2667014457237204   T   T   T
  0.8888888733972684  0.5555555468062763  0.2667014476092278   T   T   T
  0.2222222072473073  0.5555555394819613  0.2667014472298071   T   T   T
  0.5555555418348375  0.5555555431049960  0.2667014465764869   T   T   T
  0.8888888777475312  0.8888888742505837  0.2667014475198846   T   T   T
  0.2222222096896887  0.8888888778540504  0.2667014494021222   T   T   T
  0.5555555404135117  0.8888888789670559  0.2667014454419004   T   T   T
 -0.0000000093291846 -0.0000000095020838  0.3552884872196608   T   T   T
  0.3333333300228480 -0.0000000062924080  0.3552884875617840   T   T   T
  0.6666666560547367 -0.0000000072155248  0.3552884880341498   T   T   T
 -0.0000000093391197  0.3333333257297619  0.3552884864600340   T   T   T
  0.3333333309683794  0.3333333285767561  0.3552884875690537   T   T   T
  0.6666666655466773  0.3333333236851794  0.3552884870158309   T   T   T
 -0.0000000043142726  0.6666666610389311  0.3552884876976051   T   T   T
  0.3333333278712379  0.6666666585164227  0.3552884856511891   T   T   T
  0.6666666599060103  0.6666666606544034  0.3552884852609683   T   T   T
```

Ag111 (2x2x1) supercell

```
Ag
  1.00000000000000
    5.7558491988584972    0.0000000000000000    0.0000000000000000
    2.8779245994292486    4.9847116265637670    0.0000000000000000
    0.0000000000000000    0.0000000000000000   27.0494467868053334
  Ag
   16
Selective dynamics
Direct
  0.0000000000000000  0.0000000000000000  0.0850294654130223   F   F   F
  0.5000000000000000  0.0000000000000000  0.0850294654130223   F   F   F
 -0.0000000000000000  0.5000000000000000  0.0850294654130223   F   F   F
  0.4999999999999999  0.5000000000000000  0.0850294654130223   F   F   F
  0.1666666666666643  0.1666666666666643  0.1719005801578888   F   F   F
  0.6666666666666642  0.1666666666666643  0.1719005801578888   F   F   F
  0.1666666666666643  0.6666666666666643  0.1719005801578888   F   F   F
  0.6666666666666643  0.6666666666666643  0.1719005801578888   F   F   F
  0.8333333248032478  0.3333333305712949  0.2585184823258445   T   T   T
  0.3333333265956561  0.3333333251001243  0.2585184814609473   T   T   T
  0.8333333250211756  0.8333333257177051  0.2585184824075382   T   T   T
  0.3333333315144240  0.8333333204320192  0.2585184827022645   T   T   T
 -0.0000000064174232 -0.0000000013301591  0.3457906417277204   T   T   T
  0.4999999949600265 -0.0000000055976585  0.3457906423121225   T   T   T
 -0.0000000057464452  0.4999999962880696  0.3457906417202528   T   T   T
  0.4999999961758383  0.4999999945947776  0.3457906422412195   T   T   T
```

Ag111 (3x31 supercell)

```
Ag
  1.00000000000000
    8.6380164389748657    0.0000000000000000    0.0000000000000000
    4.3190082194874329    7.4807416744598259    0.0000000000000000
    0.0000000000000000    0.0000000000000000   27.0529108884204703
  Ag
   36
Selective dynamics
Direct
  0.0000000000000000  0.0000000000000000  0.1035008769129745   F   F   F
  0.3333333333333357  0.0000000000000000  0.1035008769129745   F   F   F
  0.6666666666666643  0.0000000000000000  0.1035008769129745   F   F   F
  0.0000000000000001  0.3333333333333354  0.1035008769129745   F   F   F
  0.3333333333333358  0.3333333333333354  0.1035008769129745   F   F   F
  0.6666666666666644  0.3333333333333354  0.1035008769129745   F   F   F
  0.0000000000000004  0.6666666666666634  0.1035008769129745   F   F   F
  0.3333333333333361  0.6666666666666634  0.1035008769129745   F   F   F
  0.6666666666666649  0.6666666666666634  0.1035008769129745   F   F   F
  0.1111111111111143  0.1111111111111142  0.1904035509297088   F   F   F
  0.4444444444444429  0.1111111111111142  0.1904035509297088   F   F   F
  0.7777777777777788  0.1111111111111142  0.1904035509297088   F   F   F
  0.1111111111111144  0.4444444444444425  0.1904035509297088   F   F   F
  0.4444444444444430  0.4444444444444425  0.1904035509297088   F   F   F
```

0.7777777777777788 0.4444444444444425 0.1904035509297088 F F F
0.1111111111111145 0.7777777777777780 0.1904035509297088 F F F
0.4444444444444431 0.7777777777777780 0.1904035509297088 F F F
0.7777777777777788 0.7777777777777780 0.1904035509297088 F F F
0.8888888530280930 0.2222221839578483 0.2770536779505749 T T T
0.2222221849477797 0.2222221813079360 0.2770536799860496 T T T
0.5555555166441265 0.2222221862142236 0.2770536775651041 T T T
0.8888888502984020 0.5555555196496842 0.2770536786266509 T T T
0.2222221893038395 0.5555555135006405 0.2770536802479278 T T T
0.5555555188216410 0.5555555177887050 0.2770536775595658 T T T
0.8888888521774285 0.8888888518197774 0.2770536790370572 T T T
0.2222221883633901 0.8888888521233121 0.2770536791859164 T T T
0.5555555160510776 0.8888888488956932 0.2770536784538555 T T T
-0.0000000005655671 0.0000000007793520 0.3643422874196506 T T T
0.3333333333142646 0.0000000013986913 0.3643422876774339 T T T
0.6666666638086874 0.0000000012292252 0.3643422876968596 T T T
-0.0000000021204447 0.3333333339554644 0.3643422877071616 T T T
0.3333333314303652 0.3333333322684880 0.3643422888216912 T T T
0.6666666682253886 0.3333333324757837 0.3643422888837408 T T T
-0.0000000014389592 0.6666666651267951 0.3643422876857258 T T T
0.3333333319057362 0.6666666682223712 0.3643422873277074 T T T
0.6666666684548713 0.6666666659496925 0.3643422880023126 T T T

Ru1000 (2x2x1 supercell)

Ru
1.00000000000000
5.3959999999999999 0.0000000000000000 0.0000000000000000
0.0000000000000000 4.6730730788208303 0.0000000000000000
0.0000000000000000 0.0000000000000000 30.3585000000000136
Ru
16
Selective dynamics
Direct
0.9478945918263788 0.2384671836355981 0.6996168202162413 T T T
0.4478955888836150 0.2384674258392305 0.6996167900012332 T T T
0.1980899520454035 0.7391382077831928 0.6996191603685507 T T T
0.6980893336621211 0.7391474457383422 0.6996180451927324 T T T
0.9479820951850599 0.9059572449406431 0.7675916021309903 T T T
0.4479803891105922 0.9059587366841269 0.7675919589135329 T T T
0.1981582030988638 0.4059005209307382 0.7676138995501760 T T T
0.6981566659774300 0.4058988712967258 0.7676129986656403 T T T
0.9480954665243431 0.2401140593212859 0.8377277124406068 F F F
0.4480954665243360 0.2401140593212859 0.8377277124406068 F F F
0.1980954665243431 0.7401140593212933 0.8377277124406068 F F F
0.6980954665243431 0.7401140593212933 0.8377277124406068 F F F
0.9480954665243431 0.9067807259879503 0.9075434147974484 F F F
0.4480954665243360 0.9067807259879503 0.9075434147974484 F F F
0.1980954665243431 0.4067807259879587 0.9075434147974484 F F F
0.6980954665243431 0.4067807259879587 0.9075434147974484 F F F

Co111 (2x2x1 supercell)

```
Co
  1.00000000000000
    4.9260000000000002   0.0000000000000000   0.0000000000000000
    0.0000000000000000   4.2660411390421444   0.0000000000000000
    0.0000000000000000   0.0000000000000000  35.9774999999999991
  Co
   16
Selective dynamics
Direct
  0.0010733722163123  0.3332007300715120  0.7537321292013630   T   T   T
  0.4989456208251118  0.3332204003127698  0.7537260037213500   T   T   T
  0.2499761977839875  0.8325601408864957  0.7534600658769329   T   T   T
  0.7500002873918765  0.8327607294588447  0.7524406731994943   T   T   T
 -0.0014321643310377 -0.0012174855253035  0.8088622033764065   T   T   T
  0.5014299176286391 -0.0012430472200869  0.8088647714936072   T   T   T
  0.2500112171702867  0.5016652061069025  0.8083212225618738   T   T   T
  0.7499891770443194  0.5015136210752131  0.8088718146963036   T   T   T
  0.0000000000000000  0.3333333333333354  0.8640122298658923   F   F   F
  0.5000000000000000  0.3333333333333354  0.8640122298658923   F   F   F
  0.2500000000000000  0.8333333333333350  0.8640122298658923   F   F   F
  0.7500000000000000  0.8333333333333350  0.8640122298658923   F   F   F
  0.0000000000000000  0.0000000000000000  0.9193940657355314   F   F   F
  0.5000000000000000  0.0000000000000000  0.9193940657355314   F   F   F
  0.2500000000000000  0.4999999999999996  0.9193940657355314   F   F   F
  0.7500000000000000  0.4999999999999996  0.9193940657355314   F   F   F
```

Supplementary References

1. W. A. Brown, R. Kose, D.A. King, Femtomole adsorption calorimetry on single-crystal surfaces. *Chem. Rev.* **98,** 797-831 (1998).

2. H. Froitzheim, U. Kohler, Kinetics of the Adsorption of CO on Ni(111)). *Surf. Sci.* **188,** 70-86 (1987).

3. J.B. Miller, H.R. Siddiqui, S.M. Gates, J.N. Russell, J.T. Yates, J.C. Tully, M.J. Cardillo, Extraction of Kinetic-Parameters in Temperature Programmed Desorption - a Comparison of Methods. *J. Chem. Phys.* **87**, 6725-6732 (1987).

4. J. H. Fischer-Wolfarth, J. Hartmann, J.A. Farmer, J.M. Flores-Camacho, C.T. Campbell, S. Schauermann, H. J. Freund, An improved single crystal adsorption calorimeter for determining gas adsorption and reaction energies on complex model catalysts. *Rev. Sci. Instrum* **82,** 024102 (2011).

5. X.C. Guo, J.T. Yates, Dependence of effective desorption kinetic-parameters on surface coverage and adsorption temperature – CO on Pd(111). *J. Chem. Phys.* **90**, 6761-6766 (1989).

6. J.C. Tracy, P.W. Palmberg, Structural influences on adsorbate binding energy. I. carbon monoxide on (100) Palladium. *J. Chem. Phys.***51,** 4852 (1969).

7. J. Szanyi, D.W. Goodman, CO oxidation on Palladium. 1. A combined kinetic-infrared reflection-absorption spectroscopic study of Pd(100). *J. Phys. Chem.* **98**, 2972-2977 (1994).

8. D.H. Wei, D.C. Skelton, S.D. Kevan, Desorption and molecular interactions on surfaces: CO/Rh(110), CO/Rh(100) and CO/Rh(111). *Surf. Sci.* **381,** 49-64 (1997).

9. E.G. Seebauer, A.C.F. Kong, L.D. Schmidt, Adsorption and desorption of CO and H_2 on Rh(111) - laser-induced desorption. *Appl. Surf. Sci.* **31,** 163-172 (1988).

10. P. Hollins, J. Pritchard, Interactions of co molecules adsorbed on Cu(111). *Surf. Sci.* **89**, 486-495 (1979).

11. J. Lahtinen, J. Vaari, K. Kauraala, Adsorption and structure dependent desorption of CO on Co(0001). *Surf. Sci.* **418,** 502-510 (1998).

12. R.D. Ramsier, Q. Gao, H.N. Waltenburg, K.W. Lee, O.W. Nooij, L. Lefferts, J.T. Yates, No adsorption and thermal-behavior on Pd surfaces - a detailed comparative-study. *Surf. Sci.* **320**, 209-237 (1994)

13. E.M. Karp, C.T. Campbell, F. Studt, F. Abild-Pedersen, J.K. Nørskov , Energetics of Oxygen Adatoms, Hydroxyl Species and Water Dissociation on Pt(111). *J. Phys. Chem. C*, **116** 25772-25776 (2012).

14. M. Labayen, S.A. Furman, D.A. Harrington. A thermal desorption study of iodine on Pt(111), *Surf. Sci*. **525,** 149-158. (2003).

15. K.J. Wu, S.D. Kevan, Isothermal coverage dependent measurements of NH_3 and ND_3 desorption from Cu(001). *J. Chem. Phys.* **95,** 5355-5363 (1991).

16. E.M. Karp, T.L. Silbaugh, C.T. Campbell, Energetics of Adsorbed CH_3 and CH on Pt(111) by Calorimetry: Dissociative Adsorption of CH_3I. *J. Phys. Chem. C* **117,** 6325-6336 (2013).

17. E.M. Karp, T.L. Silbaugh, M.C. Crowe, C.T. Campbell, Energetics of Adsorbed Methanol and Methoxy on Pt(111) by Microcalorimetry. *J. Am. Chem. Soc.***134,** 20388-20395. (2012).

18. H. Ihm, H.M. Ajo, J.M. Gottfried, P. Bera, C.T. Campbell, Calorimetric measurement of the heat of adsorption of benzene on Pt(111). *J. Phys. Chem. B* **108,** 14627-14633. (2004).

19. M. Xi, M.X. Yang, S.K. Jo, B.E. Bent, P. Stevens, Benzene adsorption on Cu(111) - Formation of a stable bilayer. *J. Chem. Phys.* **101**, 9122-9131 (1994).

20. W.D. Lew, M.C. Crowe, E. Karp, C.T. Campbell, Energy of Molecularly Adsorbed Water on Clean Pt(111) and Pt(111) with Coadsorbed Oxygen by Calorimetry. *J. Phys. Chem. C* **115,** 9164-9170. (2011).

21. Karp, E. M., Silbaugh, T. L. & Campbell, C. T. Energetics of adsorbed CH_3 on Pt(111) by calorimetry. *J. Am. Chem. Soc.* **135**, 5208–5211 (2013)
